# Author Correction: PHARP: a pig haplotype reference panel for genotype imputation

**DOI:** 10.1038/s41598-022-18078-y

**Published:** 2022-08-17

**Authors:** Zhen Wang, Zhenyang Zhang, Zitao Chen, Jiabao Sun, Caiyun Cao, Fen Wu, Zhong Xu, Wei Zhao, Hao Sun, Longyu Guo, Zhe Zhang, Qishan Wang, Yuchun Pan

**Affiliations:** 1 College of Animal Sciences, Zhejiang University, Hangzhou, 310058 Zhejiang China; 2 Department of Animal Science, School of Agriculture and Biology, Shanghai Jiao Tong University, Shanghai, 200240 China; 3 Hubei Key Laboratory of Animal Embryo and Molecular Breeding, Institute of Animal Husbandry and Veterinary, Hubei Provincial Academy of Agricultural Sciences, Wuhan, 430064 China; 4 Department of Animal Science, School of Animal Science, Jilin University, Changchun, 130062 China

Correction to: *Scientific Reports* https://doi.org/10.1038/s41598-022-15851-x, published online 25 July 2022

The original version of this Article contained errors in Figure 3, where plot results did not display correctly.

The original Figure 3 and accompanying legend appear below.

**Figure 3:**
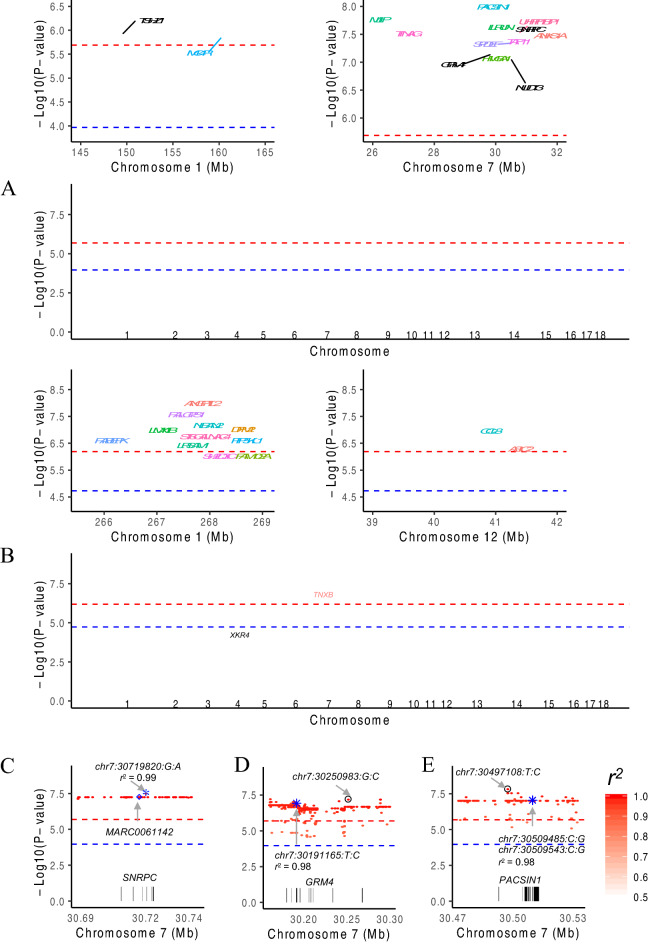
Association signals for growth phenotypes before and after imputation. Association test statistics on the − log10 (*P*-value) scale (y-axis) are plotted for each SNP position (x-axis) for the trait of backfat thickness at an age of 180 days (**A**), from Zhang et al., and at 100 kg (**B**), from Fu et al. To simplify the plot, only the variants with a *P*-value less than 1.08 × 10^−4^ are shown, and they are colored according to the annotated genes. The black-labeled genes are reported in the original paper, and the blue-labeled genes are novel genes detected after imputation. Examples of potential causal variants (marked by blue asterisks) in the *SNRPC* (**C**), *GRM4* (**D**) and *PACSIN1* (**E**) genes. Each dot represents a variant, whose LD (*r*^2^) with the Chip SNP (marked by blue diamonds) or the one with the lowest *P*-value (marked by a black circle) is indicated by the colour of the dot. The two horizontal lines divide SNPs with *P*-values < 2.05 × 10^−6^ and < 1.08 × 10^−4^ (**A**), and *P*-values < 6.46 × 10^−7^ and < 1.86 × 10^−5^ (**B**).

The original Article has been corrected.

