# Supplementary material for: PHARP: a pig haplotype reference panel for genotype imputation
Source: Sci Rep. 2022 Jul 25;12:12645. doi: 10.1038/s41598-022-15851-x (PMC9314402; doi:10.1038/s41598-022-15851-x)
Supplement: Supplementary file 1 — Supplementary Information. [file 41598_2022_15851_MOESM1_ESM.pdf]

Table S1. WGS data source for pig haplotypes reference panel construction and imputation accuracy assessment.

Type	Accession	Pig breeds	Sample size	Depth (Mean ± SD)	Ref
WGS data for PHARP construction	PHARP	AQ, DU, DY, HBBZ, JH, JS, LW, XDH	84	9.7 ± 9.3	Lab*
	PRJEB1683	EWB, AWB, DU, HA, PI, LR, LW, XZ, JQH, MS	72	6.8 ± 1.5	[1-4]
	PRJNA144099	WZS	1	82.8 ± 0	[5]
	PRJNA41185	DU	1	14.4 ± 0	-
	PRJNA176189	GM	1	46.9 ± 0	[6]
	PRJNA231897	RC	6	8.3 ± 1	-
	PRJNA186497	AWB, TWB, PZ, WJ, YN, NJ, JH	49	5.9 ± 5.9	[7]
	PRJNA238851	TC	4	7.2 ± 1.2	[8]
	PRJNA260763	DU, LR, YM, KWB, LW	70	11.2 ± 2.2	[9, 10]
	PRJEB9115	DU	1	26.4 ± 0	-
	PRJNA213179	AWB, EHL, HT, LWU, LC, MI, MX, TP, WZS	69	25.1 ± 3.1	[11]
	PRJNA221763	BK	3	4.6 ± 0.4	[12]
	PRJNA239399	MA, DU	4	13.7 ± 3	[13]
	PRJNA190683	IB	1	1.7 ± 0	-
	PRJNA255085	EWB, GC, IB, MP, LW	6	10 ± 4.9	[14]
	PRJEB9326	PI	10	10.9 ± 2.7	[15]
	PRJEB9922	EWB, AWB, WS, TP, LS, AS, BB, BK, BS, CM, CS, CA, CT, GO, HA, LB, LI, MA, MW, IB, NS, TA, RE, JQH, XZ	91	11.2 ± 3.7	[16]
	PRJNA281548	BK	10	8.9 ± 4.2	[17]
	PRJNA254936	KP	14	11.7 ± 2	[18]
	PRJNA273907	LW, LR	2	9.5 ± 1.6	-
	PRJNA438040	WZS	1	12.8 ± 0	-
	PRJNA506339	LW	16	7.8 ± 4.9	-
	PRJNA524263	WB	20	6.8 ± 1.8	-
	PRJNA369600	IB, LS	7	11.1 ± 0.4	[19]
	PRJNA418771	NS	1	34 ± 0	-
	PRJNA493166	LW	3	20.6 ± 3.3	[20]
	PRJNA487172	DU	4	11.8 ± 0.7	[21]
	PRJEB27654	GM	12	13.4 ± 0.8	[22]
	PRJNA343658	LS, DU, LW	72	8 ± 6.8	[23]
	PRJNA305975	DSE	31	5.6 ± 2.1	[24]

	PRJNA322309	RC, DU	8	16.2 ± 5.4	-
	PRJNA320525	WB, IB, ICF, YM, TA	9	11.5 ± 2.8	[25, 26]
	PRJNA354435	DP	1	24.6 ± 0	[27]
	PRJNA378496	MS, DU, AWB, DW, DSE, TP, LW, RC, MZ	71	8.5 ± 4.1	[28]
	PRJEB29465	DU, PL	5	-	-
	PRJNA507853	DLY, LL, LW, DU, LR	10	34.6 ± 2.6	[29]
	PRJNA305081	MZ, AWB	11	10 ± 1.7	-
	PRJNA291011	GM	1	14.9 ± 0	[30]
	PRJNA482384	USMARC	29	15.9 ± 1.6	[31]
	PRJNA414091	USMARC	108	17.4 ± 7.4	[31]
	PRJNA309108	BaM, BK, HA, JH, LR, LW, MS, PI, RC	9	66.4 ± 5.3	[32]
	PRJNA553106	PLL, PI, MS, LW, LL	115	5.1 ± 8.1	[33]
	PRJEB35180	Ssc	5	16.3 ± 7.9	-
	PRJNA488327	EHL, DU	19	24.3 ± 3.7	[34]
	PRJNA398176	JH, NJ, BaM, BS	24	23.8 ± 1.6	[35]
	PRJNA550237	DU, EHL, LW, TP	63	22.8 ± 3.3	[36]
	PRJEB37956	LW	5	13.9 ± 1.3	[37]
	PRJNA622908	LR	5	13.8 ± 0.9	[37]
	PRJNA626370	DU	5	32.7 ± 5.1	-
	PRJNA488960	TC, LW	12	6.9 ± 1.5	-
**Total**			**1,181**		
Test dataset 1	PRJEB39374	LW	76	17.4 ± 5.8	[37]
	PRJEB38156	LW	5	12.7 ± 1.8	[37]
**Total**			**81**		
Test dataset 2	PHARP	JXH	54	5.8 ± 1.9	Lab*
Test dataset 3	PRJNA736175	DU	299	-	Lab*
Test dataset 4	PRJNA736175	DU	20	-	Lab*

Note: * WGS data generated by our laboratory.
AQ: Anqingliubai, AS: Angler Sattleschwein, AWB: Asian wild boar, BaM: Bamei, BB: Bunte Bentheimer, BK: Berkshire, BS: Baoshan, BSB: British Saddleback, CA: Calabrese, CM: Chato Murciano, CS: Cinta Senese, CT: Casertana, DLY: Duroc x (Landrace x Yorkshire), DP: Duroc x Pietrain, DSE: Diannan small ear, DU: Duroc, DW: Daweizi, DY: Dingyuan, EHL: Erhualian, EWB: European wild boar, GC: Guatemala Creole pig, GM: Göttingen minipig, GO: Gloucester Old Spot, HA: Hampshire, HBBZ: Huibeibai, HT: Hetao, IB: Iberian, ICF: Isla del Coco feral pigs (Costa Rica), JH: Jinhua, JQH: Jiangquhai, JS: Jishen, JXH: Jiaxinghei, KP: Korean pig, KWB: Korean wild boar, LB: Large Black, LC: Luchuan, LI: Linderodsvin, LL: Landrace x Large White, LR:

Landrace, LS: Leping Spotted, LW: Large White, LWU: Laiwu, MA: Mangalica, MI: Mizhu, MP: 16th century pig, MS: Meishan, MW: Middle White, MX: Bamaxiang, MZ: Min, NJ: Neijiang, NS: Nera Siciliana, PI: Pietrain, PL: Pietrain x Swiss Landrace , PLL: Pietrain x (Landrace x Large White), PZ: Penzhou, RC: Rongchang, RE: Retinto, Ssc: Sus scrofa, TA: Tamworth, TC: Tongcheng, TP: Tibetan pig, TWB: Tibetan wild boar, USMARC: USDA-ARS-USMARC, WB: Wild boar, WJ: Wujin, WS: Wannan Spotted, WZS: Wuzhishan, XDH: Xiaduhei, XZ: Xiang, YM: Yucatan miniature pig, YN: Yanan,

Table S2. Imputation accuracy evaluated from test datasets.

Test datasets	[1]Mimic chips	No. of imputed genotypes	No. of consistent genotypes	[2]Inconsistent genotypes (percentage)				CR	r^2
				No.	Ho_Ho	He_Ho	Ho_He		
Test dataset 1 (LW, n=81)	50K	2,628,610,057	2,553,464,423	75,145,634	0.057	0.488	0.456	0.971	0.920
	60K	2,628,136,817	2,559,007,817	69,129,000	0.055	0.489	0.455	0.974	0.926
	80K	2,627,590,208	2,569,523,946	58,066,262	0.050	0.480	0.469	0.978	0.939
Test dataset 2 (JXH, n=54)	50K	1,093,333,104	884,392,699	208,940,405	0.257	0.308	0.435	0.809	0.488
	60K	1,093,163,149	891,893,395	201,269,754	0.248	0.305	0.447	0.816	0.510
	80K	1,093,001,361	893,631,949	199,369,412	0.245	0.305	0.451	0.818	0.517
Test dataset 3 (DU, n=299)	50K	2,327,943,137	2,174,512,235	153,430,902	0.041	0.503	0.456	0.934	0.876
	60K	2,327,099,658	2,185,869,172	141,230,486	0.039	0.495	0.467	0.939	0.886
	80K	2,325,327,186	2,201,309,816	124,017,370	0.036	0.492	0.472	0.947	0.901
[3]Test dataset 4 (DU, n = 20)	50K	160,966,260	149,064,448	11,901,812	0.040	0.537	0.423	0.926	0.869

Notes:

[1]Mimicing the most popular commercial porcine SNP chips (50K, 60K and 80K) by extracting SNPs for test datasets at all sites included in these commercial porcine microarray genotyping platforms.

[2]Inconsistent genotypes types: Ho_Ho, a homozygote was imputed to be another homozygote; He_Ho, a heterozygote was imputed to be a homozygote; Ho_He, a homozygote was imputed to be a heterozygote.

[3]Duroc pigs (n = 20) genotyped by 50K were used as input for imputation and the imputed-genotypes were compared with the same individuals genotyped by ELC (regarded as real).

Table S3. Imputation accuracy estimated by mimicking the imputed **panel with different densities (repeated 5 times) of SNPs on chromosomes using test datasets.**

Test datasets	Chr.	Density Kb per SNP	No. of imputed genotypes	No. of consistent genotypes	Inconsistent genotypes (percentage)				CR	r^2
					No.	Ho_Ho	He_Ho	Ho_He		
Test dataset 1	chr1	2.5	92.32±0	91.1±0.01	1.22±0.01	0.021±0	0.384±0.002	0.595±0.002	0.987±0	0.976±0
		5	96.02±0	94.58±0.01	1.44±0.01	0.023±0	0.412±0.003	0.565±0.003	0.985±0	0.973±0
		10	98.05±0	96.22±0.01	1.83±0.01	0.029±0.001	0.44±0.004	0.531±0.003	0.981±0	0.966±0
		20	99.12±0	96.55±0.04	2.57±0.04	0.036±0.002	0.471±0.003	0.493±0.002	0.974±0	0.952±0.001
		40	99.67±0	95.7±0.05	3.97±0.05	0.046±0.002	0.495±0.004	0.459±0.004	0.96±0	0.925±0.001
		50	99.78±0	95.15±0.12	4.63±0.12	0.046±0.001	0.496±0.001	0.458±0.001	0.954±0.001	0.913±0.002
		60	99.85±0	94.55±0.1	5.3±0.1	0.052±0.001	0.506±0.007	0.442±0.007	0.947±0.001	0.899±0.002
		80	99.95±0	93.64±0.1	6.31±0.1	0.056±0.003	0.503±0.005	0.44±0.006	0.937±0.001	0.88±0.002
		100	100±0	92.58±0.14	7.42±0.14	0.061±0.002	0.511±0.003	0.427±0.005	0.926±0.001	0.858±0.003
		200	100.11±0	88.17±0.23	11.94±0.23	0.079±0.001	0.525±0.002	0.396±0.001	0.881±0.002	0.768±0.004
		400	100.17±0	82.64±0.28	17.53±0.28	0.105±0.004	0.547±0.001	0.348±0.004	0.825±0.003	0.654±0.005
	chr2	2.5	61.53±0	60.56±0.02	0.97±0.02	0.022±0	0.439±0.008	0.539±0.008	0.984±0	0.972±0
		5	63.43±0	62.26±0.01	1.16±0.01	0.025±0.001	0.457±0.003	0.518±0.003	0.982±0	0.967±0
		10	64.46±0	62.92±0.01	1.53±0.01	0.03±0.001	0.477±0.006	0.493±0.005	0.976±0	0.957±0
		20	65±0	62.71±0.04	2.29±0.04	0.039±0.001	0.495±0.005	0.466±0.004	0.965±0.001	0.935±0.001
		40	65.28±0	61.59±0.04	3.69±0.04	0.049±0.002	0.511±0.005	0.44±0.005	0.943±0.001	0.894±0.001
		50	65.34±0	60.87±0.11	4.47±0.11	0.056±0.002	0.512±0.005	0.432±0.006	0.932±0.002	0.87±0.004
		60	65.37±0	60.3±0.07	5.08±0.07	0.058±0.002	0.516±0.003	0.426±0.002	0.922±0.001	0.853±0.002
		80	65.42±0	59.15±0.08	6.28±0.08	0.066±0.002	0.514±0.005	0.421±0.006	0.904±0.001	0.816±0.003
		100	65.45±0	58.24±0.15	7.21±0.15	0.067±0.004	0.517±0.002	0.416±0.004	0.89±0.002	0.79±0.004
		200	65.51±0	53.79±0.12	11.71±0.12	0.092±0.003	0.528±0.004	0.38±0.006	0.821±0.002	0.655±0.002
		400	65.54±0	48.96±0.27	16.58±0.27	0.122±0.005	0.542±0.005	0.336±0.004	0.747±0.004	0.509±0.01
	chr3	2.5	60.08±0	59.25±0	0.83±0	0.022±0.001	0.371±0.002	0.607±0.002	0.986±0	0.974±0
		5	62.02±0	60.99±0.01	1.03±0.01	0.026±0.001	0.405±0.003	0.568±0.002	0.983±0	0.969±0
		10	63.04±0	61.63±0.02	1.4±0.02	0.034±0.002	0.441±0.003	0.525±0.004	0.978±0	0.957±0.001
		20	63.57±0	61.41±0.03	2.16±0.03	0.041±0.002	0.48±0.005	0.479±0.005	0.966±0	0.934±0.001
		40	63.83±0	60.3±0.04	3.54±0.04	0.053±0.003	0.503±0.007	0.444±0.005	0.945±0.001	0.89±0.002
		50	63.89±0	59.71±0.05	4.17±0.05	0.056±0.002	0.509±0.004	0.435±0.005	0.935±0.001	0.87±0.002
		60	63.92±0	59.15±0.06	4.77±0.06	0.059±0.002	0.514±0.004	0.427±0.004	0.925±0.001	0.851±0.002

		80	63.97±0	58.2±0.1	5.76±0.1	0.065±0.001	0.524±0.005	0.411±0.004	0.91±0.002	0.82±0.003
		100	63.99±0	57.11±0.12	6.89±0.12	0.07±0.004	0.523±0.008	0.407±0.006	0.892±0.002	0.784±0.005
		200	64.05±0	53.68±0.19	10.37±0.19	0.088±0.004	0.535±0.002	0.377±0.004	0.838±0.003	0.673±0.008
		400	64.07±0	49.73±0.28	14.35±0.28	0.109±0.005	0.555±0.005	0.336±0.006	0.776±0.004	0.549±0.009
	chr4	2.5	58.82±0	58.04±0	0.79±0	0.029±0.001	0.353±0.002	0.617±0.002	0.987±0	0.975±0
		5	60.67±0	59.67±0.01	1±0.01	0.034±0.001	0.391±0.004	0.575±0.003	0.984±0	0.969±0
		10	61.66±0	60.21±0.02	1.44±0.02	0.042±0.002	0.431±0.004	0.526±0.005	0.977±0	0.955±0.001
		20	62.17±0	59.92±0.1	2.25±0.1	0.053±0.001	0.462±0.003	0.484±0.003	0.964±0.002	0.928±0.003
		40	62.43±0	58.74±0.08	3.7±0.08	0.061±0.003	0.486±0.003	0.453±0.004	0.941±0.001	0.882±0.003
		50	62.49±0	58.16±0.12	4.33±0.12	0.065±0.002	0.485±0.004	0.451±0.004	0.931±0.002	0.861±0.004
		60	62.52±0	57.63±0.14	4.89±0.14	0.067±0.004	0.496±0.005	0.437±0.006	0.922±0.002	0.843±0.005
		80	62.57±0	56.71±0.13	5.86±0.13	0.072±0.002	0.5±0.003	0.428±0.003	0.906±0.002	0.812±0.004
		100	62.59±0	55.75±0.11	6.84±0.11	0.077±0.003	0.508±0.003	0.415±0.003	0.891±0.002	0.78±0.005
		200	62.64±0	51.99±0.21	10.66±0.21	0.094±0.001	0.525±0.005	0.381±0.005	0.83±0.003	0.658±0.007
		400	62.67±0	48.05±0.22	14.62±0.22	0.117±0.003	0.547±0.006	0.336±0.008	0.767±0.003	0.533±0.007
	chr5	2.5	51.14±0	50.51±0	0.63±0	0.023±0.001	0.349±0.003	0.628±0.002	0.988±0	0.977±0
		5	52.69±0	51.92±0.01	0.77±0.01	0.026±0.001	0.382±0.004	0.592±0.004	0.985±0	0.973±0
		10	53.5±0	52.45±0.02	1.05±0.02	0.03±0.001	0.427±0.003	0.543±0.003	0.98±0	0.963±0.001
		20	53.92±0	52.25±0.03	1.67±0.03	0.037±0.002	0.47±0.003	0.493±0.004	0.969±0.001	0.941±0.001
		40	54.12±0	51.17±0.03	2.95±0.03	0.046±0.002	0.493±0.003	0.461±0.004	0.945±0.001	0.894±0.001
		50	54.17±0	50.69±0.12	3.48±0.12	0.05±0.004	0.502±0.005	0.448±0.005	0.936±0.002	0.875±0.006
		60	54.19±0	50.11±0.1	4.08±0.1	0.057±0.003	0.505±0.007	0.438±0.007	0.925±0.002	0.852±0.004
		80	54.23±0	49.14±0.09	5.09±0.09	0.062±0.002	0.51±0.004	0.429±0.004	0.906±0.002	0.815±0.004
		100	54.25±0	48.14±0.12	6.11±0.12	0.069±0.003	0.512±0.006	0.418±0.005	0.887±0.002	0.776±0.005
		200	54.29±0	44.83±0.28	9.46±0.28	0.087±0.003	0.529±0.002	0.384±0.003	0.826±0.005	0.653±0.011
		400	54.31±0	41.06±0.31	13.26±0.31	0.108±0.004	0.552±0.009	0.34±0.007	0.756±0.006	0.518±0.012
	chr6	2.5	71.2±0	70.13±0.01	1.07±0.01	0.025±0.001	0.411±0.003	0.564±0.003	0.985±0	0.972±0
		5	73.56±0	72.27±0.01	1.29±0.01	0.03±0.001	0.434±0.002	0.536±0.002	0.982±0	0.967±0
		10	74.83±0	73.15±0.02	1.67±0.02	0.035±0.002	0.46±0.005	0.505±0.006	0.978±0	0.958±0.001
		20	75.49±0	73.07±0.03	2.42±0.03	0.043±0.001	0.482±0.004	0.475±0.003	0.968±0	0.938±0.001
		40	75.82±0	72.13±0.03	3.69±0.03	0.049±0.001	0.5±0.005	0.451±0.005	0.951±0	0.906±0.001
		50	75.89±0	71.48±0.07	4.42±0.07	0.054±0.001	0.507±0.005	0.438±0.006	0.942±0.001	0.887±0.002
		60	75.94±0	70.86±0.15	5.08±0.15	0.056±0.002	0.508±0.003	0.436±0.003	0.933±0.002	0.87±0.004
		80	76±0	69.66±0.08	6.34±0.08	0.065±0.002	0.511±0.002	0.424±0.002	0.917±0.001	0.836±0.002
		100	76.03±0	68.64±0.09	7.39±0.09	0.07±0.003	0.514±0.003	0.416±0.003	0.903±0.001	0.808±0.003

		200	76.1±0	63.97±0.21	12.13±0.21	0.087±0.002	0.526±0.005	0.387±0.003	0.841±0.003	0.684±0.005
		400	76.13±0	58.49±0.29	17.64±0.29	0.109±0.002	0.54±0.003	0.351±0.005	0.768±0.004	0.543±0.007
	chr7	2.5	60.41±0	59.23±0.01	1.18±0.01	0.025±0.001	0.494±0.005	0.481±0.005	0.98±0	0.964±0
		5	62.21±0	60.8±0.02	1.41±0.02	0.029±0.001	0.51±0.004	0.461±0.004	0.977±0	0.957±0.001
		10	63.16±0	61.36±0.03	1.8±0.03	0.035±0.001	0.522±0.005	0.443±0.006	0.972±0	0.946±0.001
		20	63.64±0	61.01±0.05	2.63±0.05	0.044±0.002	0.526±0.002	0.43±0.002	0.959±0.001	0.92±0.001
		40	63.88±0	59.68±0.07	4.2±0.07	0.057±0.001	0.527±0.001	0.416±0.001	0.934±0.001	0.87±0.002
		50	63.93±0	59.03±0.09	4.9±0.09	0.064±0.001	0.528±0.005	0.408±0.005	0.923±0.001	0.847±0.003
		60	63.97±0	58.36±0.08	5.61±0.08	0.066±0.001	0.527±0.003	0.407±0.002	0.912±0.001	0.825±0.003
		80	64.01±0	57.13±0.15	6.88±0.15	0.072±0.003	0.527±0.002	0.401±0.004	0.892±0.002	0.785±0.005
		100	64.03±0	56.01±0.14	8.02±0.14	0.076±0.002	0.528±0.003	0.396±0.005	0.875±0.002	0.75±0.005
		200	64.08±0	51.96±0.34	12.12±0.34	0.096±0.001	0.543±0.003	0.361±0.004	0.811±0.005	0.622±0.009
		400	64.11±0	47.48±0.4	16.63±0.4	0.121±0.005	0.559±0.002	0.321±0.006	0.741±0.006	0.484±0.014
	chr8	2.5	66.79±0	65.94±0.01	0.85±0.01	0.024±0.001	0.345±0.002	0.631±0.003	0.987±0	0.976±0
		5	68.75±0	67.72±0.01	1.04±0.01	0.029±0	0.376±0.004	0.595±0.004	0.985±0	0.971±0
		10	69.79±0	68.38±0.02	1.41±0.02	0.035±0.001	0.414±0.005	0.551±0.005	0.98±0	0.961±0.001
		20	70.33±0	68.15±0.04	2.18±0.04	0.042±0.001	0.458±0.003	0.499±0.003	0.969±0.001	0.939±0.001
		40	70.6±0	66.94±0.07	3.66±0.07	0.053±0.003	0.486±0.005	0.461±0.004	0.948±0.001	0.897±0.002
		50	70.66±0	66.29±0.19	4.37±0.19	0.057±0.003	0.491±0.003	0.452±0.003	0.938±0.003	0.877±0.006
		60	70.69±0	65.45±0.13	5.24±0.13	0.064±0.003	0.496±0.005	0.44±0.004	0.926±0.002	0.851±0.004
		80	70.74±0	64.26±0.09	6.48±0.09	0.069±0.002	0.506±0.004	0.425±0.005	0.908±0.001	0.815±0.003
		100	70.77±0	62.88±0.3	7.89±0.3	0.075±0.004	0.511±0.002	0.413±0.004	0.889±0.004	0.774±0.01
		200	70.82±0	58.35±0.38	12.47±0.38	0.095±0.004	0.522±0.005	0.383±0.009	0.824±0.005	0.642±0.012
		400	70.85±0	53.07±0.23	17.78±0.23	0.121±0.002	0.547±0.01	0.332±0.01	0.749±0.003	0.494±0.007
	chr9	2.5	39.7±0	39.02±0.01	0.68±0.01	0.026±0.001	0.444±0.002	0.529±0.002	0.983±0	0.968±0
		5	40.76±0	39.97±0	0.8±0	0.03±0.001	0.46±0.003	0.51±0.004	0.98±0	0.963±0
		10	41.31±0	40.25±0.03	1.06±0.03	0.036±0.002	0.481±0.003	0.483±0.004	0.974±0.001	0.951±0.002
		20	41.59±0	40.03±0.03	1.56±0.03	0.045±0.003	0.502±0.003	0.453±0.004	0.962±0.001	0.927±0.002
		40	41.73±0	39.18±0.07	2.55±0.07	0.058±0.003	0.507±0.007	0.434±0.008	0.939±0.002	0.878±0.004
		50	41.76±0	38.7±0.08	3.05±0.08	0.059±0.003	0.511±0.005	0.43±0.003	0.927±0.002	0.855±0.004
		60	41.78±0	38.3±0.1	3.47±0.1	0.066±0.003	0.506±0.007	0.427±0.007	0.917±0.002	0.833±0.005
		80	41.8±0	37.43±0.08	4.37±0.08	0.074±0.004	0.511±0.006	0.416±0.008	0.896±0.002	0.789±0.004
		100	41.81±0	36.71±0.16	5.1±0.16	0.079±0.003	0.512±0.003	0.409±0.006	0.878±0.004	0.753±0.007
		200	41.84±0	34.06±0.23	7.78±0.23	0.101±0.005	0.519±0.002	0.381±0.004	0.814±0.005	0.621±0.012
		400	41.86±0	30.63±0.27	11.22±0.27	0.126±0.005	0.533±0.004	0.341±0.005	0.732±0.006	0.458±0.014

	chr10	2.5	49.44±0	48.58±0.01	0.86±0.01	0.029±0.001	0.417±0.003	0.554±0.003	0.983±0	0.967±0
		5	50.51±0	49.43±0.01	1.08±0.01	0.034±0.001	0.442±0.002	0.524±0.003	0.979±0	0.96±0
		10	51.06±0	49.53±0.03	1.53±0.03	0.044±0.001	0.469±0.002	0.486±0.002	0.97±0	0.942±0.001
		20	51.33±0	48.94±0.03	2.4±0.03	0.052±0.003	0.484±0.004	0.464±0.006	0.953±0.001	0.909±0.001
		40	51.47±0	47.38±0.1	4.1±0.1	0.066±0.003	0.5±0.002	0.435±0.004	0.92±0.002	0.842±0.005
		50	51.5±0	46.55±0.12	4.95±0.12	0.07±0.002	0.506±0.006	0.425±0.004	0.904±0.002	0.809±0.006
		60	51.52±0	45.93±0.17	5.59±0.17	0.07±0.002	0.509±0.005	0.422±0.005	0.892±0.003	0.786±0.007
		80	51.54±0	44.61±0.22	6.94±0.22	0.078±0.003	0.516±0.006	0.406±0.007	0.865±0.004	0.733±0.009
		100	51.56±0	43.41±0.3	8.15±0.3	0.086±0.002	0.512±0.005	0.401±0.004	0.842±0.006	0.685±0.011
		200	51.59±0	39.33±0.34	12.26±0.34	0.107±0.004	0.527±0.007	0.366±0.008	0.762±0.007	0.531±0.014
		400	51.6±0	35.53±0.26	16.07±0.26	0.138±0.003	0.547±0.006	0.315±0.007	0.688±0.005	0.388±0.008
	chr11	2.5	49.87±0	49.3±0	0.57±0	0.022±0.001	0.305±0.003	0.673±0.003	0.989±0	0.979±0
		5	51.1±0	50.38±0.01	0.72±0.01	0.027±0.001	0.353±0.004	0.62±0.003	0.986±0	0.973±0
		10	51.73±0	50.72±0.02	1.01±0.02	0.032±0.002	0.405±0.005	0.563±0.004	0.98±0	0.962±0.001
		20	52.05±0	50.31±0.04	1.73±0.04	0.04±0.001	0.454±0.008	0.506±0.008	0.967±0.001	0.935±0.001
		40	52.2±0	49.23±0.06	2.98±0.06	0.049±0.002	0.492±0.004	0.459±0.004	0.943±0.001	0.887±0.002
		50	52.24±0	48.67±0.07	3.57±0.07	0.055±0.003	0.5±0.005	0.446±0.004	0.932±0.001	0.864±0.003
		60	52.26±0	48.18±0.12	4.07±0.12	0.057±0.004	0.502±0.001	0.441±0.005	0.922±0.002	0.845±0.005
		80	52.28±0	47.19±0.14	5.09±0.14	0.063±0.004	0.513±0.003	0.424±0.006	0.903±0.003	0.805±0.006
		100	52.3±0	46.29±0.18	6.01±0.18	0.07±0.003	0.514±0.003	0.416±0.004	0.885±0.003	0.769±0.007
		200	52.33±0	42.9±0.32	9.44±0.32	0.092±0.005	0.535±0.005	0.373±0.008	0.82±0.006	0.635±0.013
		400	52.35±0	39.19±0.27	13.16±0.27	0.115±0.002	0.559±0.005	0.326±0.006	0.749±0.005	0.495±0.008
	chr12	2.5	34.37±0	33.7±0.01	0.67±0.01	0.029±0.001	0.413±0.003	0.559±0.004	0.981±0	0.963±0
		5	35.29±0	34.42±0.01	0.88±0.01	0.037±0.003	0.443±0.002	0.52±0.003	0.975±0	0.951±0.001
		10	35.77±0	34.51±0.02	1.26±0.02	0.045±0.003	0.473±0.005	0.483±0.006	0.965±0.001	0.93±0.002
		20	36.02±0	34.01±0.05	2.01±0.05	0.054±0.002	0.5±0.005	0.446±0.005	0.944±0.002	0.888±0.003
		40	36.14±0	32.87±0.06	3.27±0.06	0.064±0.003	0.51±0.002	0.426±0.003	0.909±0.002	0.817±0.003
		50	36.16±0	32.3±0.12	3.86±0.12	0.068±0.003	0.517±0.004	0.415±0.006	0.893±0.003	0.784±0.007
		60	36.18±0	31.77±0.1	4.41±0.1	0.071±0.004	0.52±0.004	0.409±0.007	0.878±0.003	0.755±0.007
		80	36.2±0	30.9±0.09	5.3±0.09	0.077±0.003	0.524±0.004	0.398±0.004	0.854±0.002	0.705±0.005
		100	36.21±0	30.15±0.17	6.06±0.17	0.082±0.004	0.527±0.008	0.391±0.008	0.833±0.005	0.663±0.01
		200	36.24±0	27.25±0.33	8.99±0.33	0.103±0.007	0.532±0.005	0.366±0.008	0.752±0.009	0.507±0.022
		400	36.25±0	24.52±0.23	11.73±0.23	0.13±0.007	0.548±0.004	0.321±0.01	0.677±0.006	0.363±0.015
	chr13	2.5	67.15±0	66.34±0.01	0.81±0.01	0.023±0.001	0.318±0.003	0.659±0.003	0.988±0	0.979±0
		5	69.99±0	69.04±0	0.95±0	0.027±0.001	0.342±0.002	0.631±0.003	0.986±0	0.976±0

		10	71.55±0	70.36±0.01	1.19±0.01	0.032±0.002	0.377±0.003	0.591±0.004	0.983±0	0.971±0
		20	72.36±0	70.67±0.02	1.69±0.02	0.041±0.002	0.423±0.006	0.536±0.005	0.977±0	0.958±0.001
		40	72.77±0	70.03±0.06	2.74±0.06	0.054±0.004	0.455±0.006	0.492±0.006	0.962±0.001	0.931±0.002
		50	72.86±0	69.64±0.08	3.21±0.08	0.057±0.001	0.465±0.006	0.478±0.007	0.956±0.001	0.918±0.002
		60	72.91±0	69.13±0.12	3.79±0.12	0.063±0.002	0.471±0.006	0.466±0.006	0.948±0.002	0.903±0.003
		80	72.98±0	68.15±0.14	4.83±0.14	0.072±0.005	0.477±0.005	0.451±0.007	0.934±0.002	0.874±0.005
		100	73.02±0	67.44±0.13	5.59±0.13	0.076±0.001	0.482±0.003	0.442±0.004	0.924±0.002	0.854±0.003
		200	73.11±0	63.88±0.18	9.23±0.18	0.104±0.005	0.496±0.005	0.4±0.009	0.874±0.002	0.752±0.006
		400	73.15±0	59.53±0.22	13.62±0.22	0.131±0.003	0.514±0.004	0.354±0.007	0.814±0.003	0.631±0.006
	chr14	2.5	62.71±0	61.79±0.01	0.92±0.01	0.022±0.001	0.379±0.003	0.599±0.004	0.985±0	0.973±0
		5	64.71±0	63.58±0.01	1.13±0.01	0.029±0.001	0.403±0.005	0.568±0.004	0.983±0	0.967±0
		10	65.74±0	64.25±0.01	1.5±0.01	0.036±0.001	0.438±0.004	0.526±0.004	0.977±0	0.957±0
		20	66.27±0	64.06±0.03	2.21±0.03	0.043±0.001	0.466±0.004	0.491±0.005	0.967±0	0.936±0.001
		40	66.54±0	63.06±0.11	3.48±0.11	0.052±0.002	0.489±0.005	0.459±0.005	0.948±0.002	0.898±0.003
		50	66.59±0	62.42±0.09	4.17±0.09	0.056±0.002	0.497±0.006	0.447±0.006	0.937±0.001	0.877±0.003
		60	66.62±0	61.86±0.19	4.76±0.19	0.06±0.002	0.496±0.005	0.444±0.007	0.929±0.003	0.859±0.006
		80	66.67±0	60.72±0.18	5.95±0.18	0.067±0.002	0.505±0.005	0.428±0.006	0.911±0.003	0.822±0.006
		100	66.7±0	59.97±0.06	6.73±0.06	0.071±0.002	0.512±0.006	0.417±0.007	0.899±0.001	0.798±0.002
		200	66.75±0	56.05±0.22	10.7±0.22	0.088±0.003	0.529±0.006	0.383±0.005	0.84±0.003	0.68±0.007
		400	66.78±0	51.48±0.3	15.3±0.3	0.116±0.001	0.549±0.006	0.335±0.007	0.771±0.005	0.54±0.007
	chr15	2.5	52.16±0	51.48±0	0.68±0	0.018±0	0.393±0.004	0.588±0.003	0.987±0	0.977±0
		5	53.99±0	53.19±0.01	0.8±0.01	0.02±0.001	0.417±0.003	0.563±0.004	0.985±0	0.974±0
		10	55.01±0	53.99±0.01	1.02±0.01	0.023±0.001	0.443±0.002	0.535±0.002	0.981±0	0.967±0.001
		20	55.55±0	54.03±0.03	1.52±0.03	0.029±0.002	0.477±0.006	0.494±0.007	0.973±0.001	0.95±0.001
		40	55.83±0	53.32±0.04	2.51±0.04	0.036±0.002	0.501±0.004	0.463±0.003	0.955±0.001	0.918±0.001
		50	55.89±0	52.84±0.1	3.05±0.1	0.041±0.003	0.507±0.004	0.452±0.005	0.945±0.002	0.899±0.004
		60	55.93±0	52.37±0.09	3.56±0.09	0.042±0.001	0.515±0.006	0.443±0.006	0.936±0.002	0.883±0.003
		80	55.97±0	51.52±0.11	4.46±0.11	0.048±0.001	0.52±0.008	0.432±0.009	0.92±0.002	0.852±0.004
		100	56±0	50.7±0.2	5.31±0.2	0.052±0.002	0.523±0.005	0.425±0.006	0.905±0.004	0.824±0.007
		200	56.06±0	47.01±0.22	9.05±0.22	0.077±0.002	0.532±0.005	0.391±0.007	0.839±0.004	0.694±0.007
		400	56.09±0	43.17±0.29	12.92±0.29	0.105±0.003	0.554±0.006	0.341±0.008	0.77±0.005	0.56±0.01
	chr16	2.5	40.93±0	40.42±0.01	0.52±0.01	0.027±0.001	0.319±0.001	0.654±0.001	0.987±0	0.978±0
		5	42.04±0	41.4±0.01	0.64±0.01	0.033±0.001	0.356±0.004	0.611±0.006	0.985±0	0.973±0
		10	42.64±0	41.76±0.02	0.88±0.02	0.039±0.001	0.396±0.004	0.565±0.004	0.979±0	0.963±0.001
		20	42.95±0	41.57±0.05	1.39±0.05	0.052±0.005	0.436±0.004	0.513±0.003	0.968±0.001	0.941±0.003

		40	43.11±0	40.8±0.05	2.31±0.05	0.063±0.002	0.467±0.002	0.47±0.003	0.946±0.001	0.901±0.002
		50	43.15±0	40.32±0.1	2.83±0.1	0.072±0.004	0.476±0.007	0.452±0.008	0.935±0.002	0.877±0.005
		60	43.17±0	39.86±0.09	3.31±0.09	0.073±0.001	0.489±0.009	0.439±0.01	0.923±0.002	0.856±0.004
		80	43.19±0	39.01±0.22	4.19±0.22	0.081±0.007	0.494±0.006	0.425±0.005	0.903±0.005	0.817±0.012
		100	43.21±0	38.34±0.1	4.87±0.1	0.084±0.002	0.494±0.004	0.422±0.006	0.887±0.002	0.788±0.005
		200	43.24±0	35.61±0.11	7.63±0.11	0.104±0.006	0.508±0.003	0.388±0.007	0.824±0.002	0.667±0.007
		400	43.26±0	32.23±0.26	11.03±0.26	0.127±0.002	0.522±0.006	0.351±0.006	0.745±0.006	0.525±0.011
	chr17	2.5	35.53±0	34.97±0.01	0.56±0.01	0.032±0.002	0.402±0.004	0.566±0.005	0.984±0	0.971±0
		5	36.47±0	35.78±0.01	0.69±0.01	0.042±0.002	0.426±0.007	0.532±0.007	0.981±0	0.964±0.001
		10	36.97±0	35.99±0.02	0.97±0.02	0.054±0.004	0.457±0.005	0.489±0.004	0.974±0	0.948±0.001
		20	37.22±0	35.77±0.04	1.45±0.04	0.061±0.005	0.477±0.003	0.462±0.007	0.961±0.001	0.923±0.003
		40	37.35±0	34.92±0.03	2.42±0.03	0.068±0.003	0.497±0.007	0.434±0.009	0.935±0.001	0.871±0.002
		50	37.37±0	34.48±0.07	2.89±0.07	0.072±0.001	0.499±0.007	0.429±0.007	0.923±0.002	0.846±0.004
		60	37.39±0	34.14±0.05	3.25±0.05	0.076±0.002	0.506±0.005	0.418±0.005	0.913±0.001	0.826±0.003
		80	37.41±0	33.32±0.14	4.09±0.14	0.08±0.004	0.511±0.008	0.409±0.009	0.891±0.004	0.782±0.007
		100	37.42±0	32.69±0.14	4.73±0.14	0.082±0.003	0.51±0.002	0.408±0.003	0.874±0.004	0.75±0.007
		200	37.45±0	29.81±0.12	7.64±0.12	0.099±0.002	0.524±0.004	0.377±0.005	0.796±0.003	0.601±0.007
		400	37.46±0	26.25±0.31	11.21±0.31	0.122±0.004	0.536±0.008	0.341±0.006	0.701±0.008	0.429±0.016
	chr18	2.5	26.11±0	25.75±0	0.36±0	0.022±0.001	0.394±0.005	0.584±0.005	0.986±0	0.975±0
		5	26.88±0	26.44±0.01	0.44±0.01	0.024±0.001	0.422±0.006	0.554±0.006	0.984±0	0.97±0.001
		10	27.29±0	26.71±0.01	0.58±0.01	0.027±0.001	0.456±0.005	0.517±0.005	0.979±0	0.96±0.001
		20	27.51±0	26.62±0.01	0.89±0.01	0.033±0.001	0.49±0.006	0.477±0.006	0.968±0.001	0.939±0.001
		40	27.63±0	26.07±0.04	1.55±0.04	0.041±0.004	0.52±0.007	0.439±0.004	0.944±0.001	0.893±0.003
		50	27.65±0	25.73±0.05	1.92±0.05	0.046±0.004	0.523±0.007	0.431±0.007	0.931±0.002	0.867±0.005
		60	27.66±0	25.39±0.17	2.28±0.17	0.053±0.004	0.525±0.011	0.422±0.011	0.918±0.006	0.84±0.012
		80	27.68±0	24.97±0.09	2.71±0.09	0.056±0.003	0.528±0.01	0.416±0.011	0.902±0.003	0.81±0.006
		100	27.69±0	24.37±0.11	3.33±0.11	0.063±0.003	0.525±0.005	0.413±0.003	0.88±0.004	0.766±0.008
		200	27.72±0	22.59±0.14	5.13±0.14	0.088±0.005	0.533±0.004	0.379±0.005	0.815±0.005	0.633±0.012
		400	27.73±0	20.48±0.2	7.25±0.2	0.121±0.005	0.549±0.008	0.33±0.01	0.739±0.007	0.477±0.013
Test dataset 2	chr1	2.5	32.72±0	27.94±0.01	4.78±0.01	0.135±0.001	0.296±0.001	0.569±0.001	0.854±0	0.73±0.001
		5	34.34±0	28.75±0.02	5.59±0.02	0.144±0.001	0.32±0.002	0.536±0.002	0.837±0	0.697±0.001
		10	35.25±0	28.7±0.02	6.55±0.02	0.156±0	0.34±0.001	0.504±0.001	0.814±0.001	0.65±0.001
		20	35.76±0	28.01±0.04	7.75±0.04	0.173±0.002	0.355±0.003	0.472±0.004	0.783±0.001	0.586±0.003
		40	36.05±0	26.95±0.04	9.1±0.04	0.198±0.001	0.359±0.003	0.444±0.004	0.748±0.001	0.509±0.002
		50	36.11±0	26.51±0.1	9.61±0.1	0.205±0.002	0.363±0.002	0.432±0.003	0.734±0.003	0.481±0.005

		60	36.16±0	26.09±0.04	10.07±0.04	0.214±0.003	0.361±0.002	0.425±0.004	0.722±0.001	0.454±0.003
		80	36.22±0	25.48±0.01	10.73±0.01	0.225±0.004	0.363±0.001	0.412±0.004	0.704±0	0.417±0.003
		100	36.25±0	24.97±0.09	11.28±0.09	0.234±0.002	0.366±0.002	0.401±0.004	0.689±0.003	0.387±0.005
		200	36.32±0	23.26±0.03	13.07±0.03	0.266±0.003	0.365±0.003	0.369±0.006	0.64±0.001	0.289±0.002
	chr2	2.5	22.88±0	19.07±0.01	3.82±0.01	0.132±0.001	0.327±0.002	0.541±0.003	0.833±0.001	0.681±0.001
		5	23.85±0	19.31±0.03	4.54±0.03	0.145±0.001	0.351±0.002	0.504±0.003	0.809±0.001	0.631±0.003
		10	24.39±0	18.92±0.04	5.47±0.04	0.164±0.001	0.37±0.002	0.466±0.002	0.776±0.002	0.561±0.003
		20	24.69±0	18.11±0.02	6.57±0.02	0.187±0.001	0.38±0.001	0.433±0.002	0.734±0.001	0.473±0.002
		40	24.85±0	16.99±0.09	7.86±0.09	0.213±0.002	0.384±0.004	0.402±0.005	0.684±0.004	0.372±0.007
		50	24.89±0	16.55±0.05	8.33±0.05	0.222±0.002	0.386±0.002	0.392±0.004	0.665±0.002	0.337±0.005
		60	24.91±0	16.33±0.06	8.58±0.06	0.227±0.003	0.39±0.003	0.383±0.003	0.656±0.002	0.32±0.006
		80	24.94±0	15.87±0.08	9.07±0.08	0.236±0.002	0.389±0.002	0.375±0.003	0.636±0.003	0.284±0.006
		100	24.96±0	15.58±0.08	9.38±0.08	0.244±0.003	0.392±0.002	0.365±0.004	0.624±0.003	0.261±0.003
		200	25±0	14.7±0.11	10.3±0.11	0.268±0.006	0.398±0.003	0.334±0.006	0.588±0.005	0.196±0.011
	chr3	2.5	24.47±10.3	21.2±8.88	3.27±1.42	0.115±0.002	0.277±0.004	0.608±0.002	0.868±0.006	0.754±0.008
		5	25.31±10.69	21.44±9.04	3.87±1.65	0.128±0.005	0.313±0.006	0.559±0.011	0.848±0.002	0.711±0.004
		10	30.64±0	25.09±0.03	5.55±0.03	0.142±0.002	0.342±0.001	0.516±0.001	0.819±0.001	0.655±0.002
		20	30.94±0	24.19±0.03	6.75±0.03	0.16±0.001	0.367±0.003	0.473±0.005	0.782±0.001	0.58±0.002
		40	31.1±0	22.89±0.04	8.21±0.04	0.185±0.002	0.379±0.003	0.436±0.002	0.736±0.001	0.486±0.004
		50	31.13±0	22.47±0.11	8.66±0.11	0.193±0.004	0.382±0.004	0.425±0.003	0.722±0.003	0.456±0.009
		60	31.15±0	22.07±0.07	9.09±0.07	0.199±0.003	0.388±0.003	0.413±0.002	0.708±0.002	0.431±0.006
		80	31.18±0	21.42±0.12	9.76±0.12	0.21±0.003	0.391±0.003	0.399±0.003	0.687±0.004	0.389±0.009
		100	31.2±0	20.93±0.17	10.27±0.17	0.218±0.006	0.391±0.002	0.39±0.006	0.671±0.006	0.358±0.013
		200	31.23±0	19.38±0.03	11.85±0.03	0.247±0.007	0.396±0.002	0.358±0.008	0.62±0.001	0.264±0.005
	chr4	2.5	25.6±0	21.41±0.01	4.19±0.01	0.128±0.001	0.294±0.001	0.579±0.002	0.836±0	0.687±0.001
		5	23.75±6.3	19.37±5.16	4.38±1.14	0.142±0.003	0.315±0.002	0.542±0.003	0.815±0.002	0.64±0.006
		10	27.09±0	21.36±0.05	5.73±0.05	0.158±0.003	0.334±0.001	0.507±0.003	0.788±0.002	0.583±0.006
		20	27.37±0	20.54±0.05	6.83±0.05	0.179±0.002	0.349±0.001	0.471±0.002	0.75±0.002	0.501±0.004
		40	27.53±0	19.46±0.03	8.07±0.03	0.199±0.004	0.36±0.004	0.441±0.006	0.707±0.001	0.414±0.004
		50	27.56±0	19.05±0.05	8.51±0.05	0.207±0.003	0.361±0.001	0.432±0.004	0.691±0.002	0.381±0.005
		60	27.58±0	18.73±0.07	8.85±0.07	0.213±0.004	0.364±0.004	0.423±0.005	0.679±0.002	0.357±0.007
		80	27.61±0	18.18±0.05	9.44±0.05	0.222±0.005	0.364±0.003	0.414±0.006	0.658±0.002	0.317±0.006
		100	27.63±0	17.79±0.06	9.84±0.06	0.226±0.004	0.368±0.003	0.406±0.004	0.644±0.002	0.293±0.006
		200	27.66±0	16.63±0.11	11.03±0.11	0.249±0.004	0.375±0.002	0.376±0.005	0.601±0.004	0.216±0.008
	chr5	2.5	19.68±0	16.54±0.01	3.13±0.01	0.138±0.002	0.299±0.002	0.563±0.003	0.841±0.001	0.693±0.002

		5	17.5±6.56	14.29±5.32	3.2±1.24	0.159±0.008	0.329±0.003	0.512±0.011	0.818±0.005	0.641±0.006
		10	20.84±0	16.31±0.02	4.53±0.02	0.175±0.002	0.351±0.003	0.474±0.004	0.782±0.001	0.567±0.002
		20	21.07±0	15.53±0.04	5.54±0.04	0.201±0.002	0.361±0.001	0.438±0.003	0.737±0.002	0.47±0.005
		40	21.19±0	14.52±0.05	6.67±0.05	0.228±0.004	0.368±0.003	0.404±0.003	0.685±0.002	0.364±0.007
		50	21.22±0	14.24±0.03	6.98±0.03	0.233±0.003	0.37±0.002	0.397±0.002	0.671±0.001	0.338±0.002
		60	21.23±0	13.93±0.06	7.3±0.06	0.239±0.002	0.367±0.003	0.393±0.004	0.656±0.003	0.31±0.006
		80	21.26±0	13.48±0.09	7.77±0.09	0.25±0.001	0.369±0.003	0.381±0.002	0.634±0.004	0.27±0.007
		100	21.27±0	13.1±0.03	8.17±0.03	0.259±0.005	0.37±0.004	0.371±0.009	0.616±0.001	0.236±0.005
		200	21.3±0	12.22±0.1	9.08±0.1	0.28±0.006	0.372±0.003	0.348±0.007	0.574±0.005	0.166±0.008
	chr6	2.5	31.75±0	27.01±0.01	4.74±0.01	0.122±0.001	0.29±0.002	0.588±0.002	0.851±0	0.718±0.001
		5	33.04±0	27.47±0.02	5.57±0.02	0.135±0.001	0.316±0.002	0.549±0.002	0.831±0.001	0.677±0.001
		10	33.75±0	27.08±0.01	6.67±0.01	0.157±0.001	0.339±0.001	0.503±0.001	0.802±0	0.611±0.001
		20	34.13±0	26.07±0.04	8.06±0.04	0.179±0.002	0.357±0.003	0.464±0.004	0.764±0.001	0.529±0.004
		40	34.33±0	24.58±0.08	9.74±0.08	0.206±0.003	0.368±0.004	0.426±0.006	0.716±0.002	0.429±0.005
		50	34.37±0	24.09±0.16	10.28±0.16	0.213±0.003	0.372±0.003	0.415±0.004	0.701±0.005	0.399±0.01
		60	34.4±0	23.64±0.09	10.76±0.09	0.221±0.002	0.374±0.003	0.405±0.002	0.687±0.003	0.371±0.006
		80	34.44±0	22.88±0.05	11.56±0.05	0.233±0.002	0.374±0.003	0.394±0.003	0.664±0.001	0.326±0.002
		100	34.46±0	22.3±0.07	12.16±0.07	0.24±0.002	0.376±0.002	0.384±0.003	0.647±0.002	0.295±0.005
		200	34.5±0	20.74±0.09	13.77±0.09	0.265±0.002	0.382±0.003	0.353±0.003	0.601±0.003	0.212±0.005
	chr7	2.5	26.81±0	22.63±0.01	4.18±0.01	0.12±0.001	0.298±0.003	0.581±0.003	0.844±0	0.707±0.001
		5	27.74±0	22.88±0.01	4.86±0.01	0.132±0.001	0.325±0.001	0.543±0.002	0.825±0	0.667±0.002
		10	28.24±0	22.54±0.02	5.7±0.02	0.147±0.002	0.348±0.002	0.504±0.004	0.798±0.001	0.611±0.001
		20	28.5±0	21.78±0.04	6.73±0.04	0.163±0.002	0.369±0.002	0.469±0.003	0.764±0.001	0.542±0.003
		40	28.65±0	20.65±0.04	7.99±0.04	0.188±0.002	0.381±0.002	0.431±0.003	0.721±0.002	0.452±0.004
		50	28.68±0	20.33±0.03	8.34±0.03	0.19±0.004	0.386±0.002	0.424±0.002	0.709±0.001	0.432±0.004
		60	28.7±0	19.94±0.11	8.76±0.11	0.202±0.004	0.385±0.003	0.414±0.005	0.695±0.004	0.4±0.008
		80	28.72±0	19.46±0.11	9.26±0.11	0.21±0.005	0.387±0.002	0.403±0.007	0.677±0.004	0.367±0.009
		100	28.74±0	19.03±0.1	9.71±0.1	0.219±0.004	0.389±0.002	0.392±0.004	0.662±0.004	0.336±0.003
		200	28.77±0	17.81±0.04	10.96±0.04	0.244±0.004	0.396±0.004	0.36±0.006	0.619±0.001	0.256±0.004
	chr8	2.5	21.64±0	17.59±0.01	4.05±0.01	0.126±0.001	0.321±0.001	0.552±0.001	0.813±0	0.633±0.001
		5	22.44±0	17.63±0.01	4.8±0.01	0.137±0	0.347±0.002	0.516±0.002	0.786±0.001	0.578±0.001
		10	22.88±0	17.22±0.03	5.66±0.03	0.15±0	0.364±0.002	0.487±0.002	0.753±0.001	0.511±0.002
		20	23.13±0	16.46±0.03	6.67±0.03	0.164±0.001	0.374±0.003	0.462±0.003	0.712±0.001	0.43±0.002
		40	23.27±0	15.54±0.06	7.73±0.06	0.181±0.002	0.383±0.002	0.437±0.001	0.668±0.003	0.346±0.006
		50	23.3±0	15.19±0.13	8.11±0.13	0.188±0.002	0.385±0.003	0.427±0.003	0.652±0.006	0.317±0.01

		60	23.32±0	14.99±0.04	8.33±0.04	0.192±0.002	0.388±0.001	0.42±0.002	0.643±0.002	0.299±0.003
		80	23.35±0	14.51±0.05	8.84±0.05	0.202±0.002	0.388±0.002	0.409±0.004	0.622±0.002	0.259±0.004
		100	23.37±0	14.25±0.07	9.11±0.07	0.208±0.003	0.392±0.001	0.399±0.004	0.61±0.003	0.239±0.007
		200	23.4±0	13.48±0.02	9.92±0.02	0.229±0.004	0.402±0.002	0.368±0.004	0.576±0.001	0.177±0.004
	chr9	2.5	15.77±0	13.14±0.01	2.63±0	0.126±0.001	0.31±0.001	0.564±0.002	0.833±0	0.683±0.001
		5	16.3±0	13.17±0.01	3.13±0.01	0.14±0.001	0.339±0.002	0.52±0.003	0.808±0.001	0.631±0.002
		10	16.58±0	12.85±0.03	3.73±0.03	0.158±0.003	0.36±0.003	0.482±0.005	0.775±0.002	0.562±0.004
		20	16.74±0	12.28±0.03	4.46±0.03	0.178±0.001	0.376±0.002	0.447±0.002	0.733±0.002	0.478±0.002
		40	16.82±0	11.57±0.09	5.25±0.09	0.202±0.005	0.382±0.002	0.416±0.005	0.688±0.005	0.386±0.013
		50	16.83±0	11.37±0.07	5.46±0.07	0.208±0.003	0.387±0.003	0.405±0.007	0.675±0.004	0.361±0.008
		60	16.85±0	11.19±0.03	5.66±0.03	0.212±0.004	0.387±0.004	0.402±0.008	0.664±0.002	0.341±0.003
		80	16.86±0	10.91±0.07	5.95±0.07	0.221±0.006	0.389±0.004	0.39±0.005	0.647±0.004	0.309±0.009
		100	16.87±0	10.66±0.05	6.21±0.05	0.231±0.005	0.388±0.002	0.381±0.006	0.632±0.003	0.278±0.007
		200	16.89±0	10.09±0.1	6.8±0.1	0.247±0.011	0.394±0.004	0.358±0.014	0.597±0.006	0.22±0.013
	chcr10	2.5	18.69±0	15.48±0.01	3.21±0.01	0.137±0.001	0.306±0.001	0.557±0.002	0.828±0.001	0.674±0.002
		5	19.2±0	15.42±0.01	3.79±0.01	0.152±0.001	0.33±0.001	0.518±0.001	0.803±0	0.621±0.001
		10	19.48±0	14.89±0.03	4.58±0.03	0.174±0.002	0.349±0.002	0.476±0.003	0.765±0.002	0.54±0.004
		20	19.62±0	14.07±0.05	5.55±0.05	0.2±0.004	0.36±0.003	0.439±0.005	0.717±0.003	0.441±0.006
		40	19.7±0	13.15±0.06	6.55±0.06	0.228±0.006	0.367±0.002	0.405±0.007	0.668±0.003	0.341±0.009
		50	19.71±0	12.81±0.05	6.9±0.05	0.238±0.005	0.366±0.002	0.397±0.005	0.65±0.003	0.306±0.008
		60	19.72±0	12.55±0.05	7.17±0.05	0.244±0.003	0.368±0.003	0.388±0.006	0.636±0.003	0.282±0.005
		80	19.73±0	12.17±0.03	7.56±0.03	0.253±0.004	0.368±0.002	0.379±0.005	0.617±0.001	0.247±0.002
		100	19.74±0	11.91±0.04	7.83±0.04	0.261±0.005	0.371±0.002	0.368±0.006	0.603±0.002	0.223±0.004
		200	19.76±0	11.23±0.04	8.53±0.04	0.284±0.005	0.379±0.002	0.337±0.007	0.568±0.002	0.163±0.006
	chr11	2.5	18.79±0	15.79±0.01	3±0.01	0.129±0.001	0.287±0.002	0.584±0.002	0.84±0.001	0.689±0.001
		5	19.35±0	15.81±0.01	3.54±0.01	0.145±0.001	0.317±0.002	0.538±0.003	0.817±0	0.638±0.001
		10	19.64±0	15.42±0.03	4.23±0.03	0.165±0.004	0.344±0.003	0.491±0.005	0.785±0.001	0.568±0.005
		20	19.8±0	14.74±0.02	5.06±0.02	0.188±0.002	0.364±0.003	0.448±0.002	0.744±0.001	0.481±0.004
		40	19.89±0	13.87±0.12	6.02±0.12	0.211±0.004	0.375±0.003	0.414±0.005	0.697±0.006	0.386±0.012
		50	19.91±0	13.47±0.1	6.43±0.1	0.22±0.005	0.378±0.004	0.402±0.004	0.677±0.005	0.346±0.01
		60	19.92±0	13.24±0.09	6.68±0.09	0.228±0.006	0.38±0.002	0.392±0.004	0.664±0.005	0.321±0.012
		80	19.94±0	12.83±0.11	7.11±0.11	0.237±0.006	0.384±0.005	0.379±0.008	0.643±0.006	0.283±0.013
		100	19.95±0	12.63±0.06	7.32±0.06	0.244±0.006	0.385±0.005	0.371±0.009	0.633±0.003	0.263±0.008
		200	19.97±0	11.7±0.05	8.26±0.05	0.267±0.003	0.397±0.006	0.337±0.008	0.586±0.002	0.185±0.005
	chr12	2.5	15.74±0	13.48±0.01	2.26±0.01	0.126±0.002	0.306±0.001	0.568±0.002	0.856±0	0.725±0.001

		5	16.27±0	13.46±0.02	2.81±0.02	0.146±0.002	0.344±0.003	0.51±0.004	0.827±0.001	0.663±0.003
		10	16.55±0	13.06±0.02	3.49±0.02	0.166±0.001	0.375±0.002	0.459±0.003	0.789±0.001	0.584±0.002
		20	16.7±0	12.32±0.07	4.39±0.07	0.192±0.002	0.392±0.003	0.416±0.002	0.737±0.004	0.48±0.008
		40	16.78±0	11.43±0.09	5.35±0.09	0.218±0.005	0.398±0.004	0.384±0.005	0.681±0.005	0.371±0.011
		50	16.8±0	11.17±0.07	5.62±0.07	0.22±0.002	0.4±0.003	0.38±0.003	0.665±0.004	0.347±0.008
		60	16.81±0	10.85±0.11	5.95±0.11	0.233±0.007	0.405±0.002	0.362±0.005	0.646±0.007	0.309±0.015
		80	16.82±0	10.52±0.07	6.31±0.07	0.239±0.003	0.399±0.002	0.362±0.003	0.625±0.004	0.274±0.007
		100	16.83±0	10.2±0.08	6.63±0.08	0.251±0.006	0.404±0.006	0.345±0.008	0.606±0.005	0.241±0.011
		200	16.85±0	9.31±0.15	7.54±0.15	0.276±0.005	0.398±0.004	0.326±0.006	0.553±0.009	0.157±0.014
	chr13	2.5	16.25±0	13.45±0.01	2.8±0.01	0.139±0.001	0.321±0.002	0.54±0.002	0.828±0.001	0.671±0.001
		5	17.18±0	13.86±0.02	3.33±0.02	0.153±0	0.34±0.002	0.507±0.002	0.806±0.001	0.626±0.002
		10	17.73±0	13.82±0.03	3.92±0.03	0.169±0.003	0.354±0.002	0.476±0.003	0.779±0.001	0.568±0.004
		20	18.05±0	13.51±0.04	4.54±0.04	0.186±0.002	0.361±0.001	0.453±0.003	0.749±0.002	0.504±0.006
		40	18.24±0	13±0.02	5.24±0.02	0.206±0.004	0.367±0.001	0.428±0.004	0.713±0.001	0.43±0.005
		50	18.28±0	12.78±0.04	5.5±0.04	0.21±0.002	0.368±0.001	0.422±0.002	0.699±0.002	0.405±0.005
		60	18.31±0	12.51±0.04	5.8±0.04	0.222±0.004	0.366±0.004	0.412±0.005	0.683±0.002	0.369±0.007
		80	18.35±0	12.22±0.05	6.13±0.05	0.233±0.002	0.37±0.002	0.397±0.003	0.666±0.003	0.334±0.006
		100	18.37±0	11.99±0.05	6.39±0.05	0.239±0.005	0.371±0.002	0.39±0.005	0.652±0.002	0.309±0.007
	chr14	2.5	28.26±0	23.98±0.01	4.27±0.01	0.13±0.001	0.288±0.002	0.582±0.002	0.849±0	0.708±0.002
		5	29.34±0	24.31±0.02	5.03±0.02	0.144±0.001	0.315±0.001	0.541±0.002	0.829±0.001	0.663±0.002
		10	29.93±0	23.96±0.03	5.96±0.03	0.165±0.002	0.337±0.003	0.498±0.003	0.801±0.001	0.6±0.003
		20	30.24±0	23.09±0.05	7.15±0.05	0.187±0.003	0.354±0.002	0.459±0.004	0.764±0.002	0.519±0.005
		40	30.41±0	22±0.05	8.41±0.05	0.209±0.003	0.366±0.003	0.425±0.003	0.724±0.001	0.434±0.005
		50	30.45±0	21.52±0.06	8.93±0.06	0.218±0.003	0.37±0.001	0.412±0.004	0.707±0.002	0.399±0.005
		60	30.47±0	21.19±0.14	9.28±0.14	0.22±0.003	0.371±0.003	0.409±0.003	0.695±0.005	0.379±0.011
		80	30.5±0	20.65±0.07	9.85±0.07	0.229±0.001	0.374±0.001	0.397±0.003	0.677±0.002	0.343±0.004
		100	30.52±0	20.17±0.09	10.35±0.09	0.237±0.001	0.375±0.003	0.388±0.002	0.661±0.003	0.311±0.006
		200	30.56±0	18.91±0.15	11.65±0.15	0.259±0.004	0.389±0.003	0.352±0.006	0.619±0.005	0.235±0.009
	chr15	2.5	20.08±0	16.89±0.01	3.19±0.01	0.135±0.001	0.304±0.002	0.561±0.003	0.841±0.001	0.702±0.002
		5	20.92±0	17.18±0.02	3.74±0.02	0.15±0.001	0.326±0.002	0.524±0.003	0.821±0.001	0.659±0.002
		10	21.39±0	16.98±0.03	4.41±0.03	0.168±0.003	0.345±0.001	0.487±0.004	0.794±0.002	0.599±0.004
		20	21.65±0	16.45±0.02	5.2±0.02	0.187±0.003	0.355±0.003	0.459±0.003	0.76±0.001	0.528±0.003
		40	21.8±0	15.76±0.04	6.04±0.04	0.206±0.003	0.363±0.003	0.431±0.002	0.723±0.002	0.452±0.005
		50	21.83±0	15.42±0.05	6.4±0.05	0.215±0.003	0.363±0.002	0.422±0.005	0.707±0.002	0.419±0.006
		60	21.85±0	15.13±0.07	6.72±0.07	0.223±0.003	0.362±0.004	0.414±0.005	0.693±0.003	0.39±0.007

		80	21.88±0	14.81±0.07	7.07±0.07	0.228±0.004	0.364±0.004	0.407±0.006	0.677±0.003	0.362±0.007
		100	21.9±0	14.46±0.08	7.44±0.08	0.24±0.002	0.367±0.002	0.393±0.003	0.66±0.004	0.328±0.007
		200	21.93±0	13.59±0.15	8.35±0.15	0.262±0.005	0.369±0.005	0.369±0.004	0.62±0.007	0.252±0.011
	chr16	2.5	11.47±0	9.66±0.01	1.81±0.01	0.14±0.002	0.29±0.001	0.571±0.002	0.842±0.001	0.692±0.002
		5	11.92±0	9.72±0.01	2.2±0.01	0.16±0.002	0.321±0.001	0.519±0.003	0.816±0.001	0.632±0.002
		10	12.16±0	9.49±0.04	2.68±0.04	0.183±0.005	0.344±0.003	0.473±0.004	0.78±0.003	0.554±0.009
		20	12.3±0	9.04±0.03	3.26±0.03	0.211±0.002	0.353±0.003	0.436±0.003	0.735±0.003	0.456±0.004
		40	12.37±0	8.51±0.07	3.87±0.07	0.239±0.005	0.358±0.004	0.403±0.009	0.688±0.005	0.355±0.011
		50	12.39±0	8.36±0.04	4.03±0.04	0.248±0.003	0.364±0.003	0.387±0.005	0.675±0.003	0.328±0.006
		60	12.4±0	8.23±0.03	4.17±0.03	0.254±0.006	0.362±0.003	0.384±0.007	0.664±0.003	0.306±0.007
		80	12.42±0	8.01±0.05	4.41±0.05	0.26±0.005	0.366±0.004	0.373±0.007	0.645±0.004	0.274±0.007
		100	12.42±0	7.84±0.06	4.59±0.06	0.271±0.005	0.364±0.001	0.365±0.005	0.631±0.005	0.244±0.01
		200	12.44±0	7.36±0.04	5.08±0.04	0.29±0.007	0.369±0.005	0.341±0.007	0.592±0.003	0.179±0.008
	chr17	2.5	15.04±0	12.79±0.01	2.25±0.01	0.12±0.001	0.293±0.002	0.587±0.002	0.85±0	0.718±0.001
		5	15.55±0	12.93±0.01	2.62±0.01	0.13±0.002	0.322±0.001	0.548±0.002	0.832±0.001	0.68±0.003
		10	15.82±0	12.7±0.01	3.11±0.01	0.144±0.001	0.351±0.002	0.505±0.003	0.803±0.001	0.622±0.002
		20	15.96±0	12.2±0.03	3.76±0.03	0.16±0.002	0.371±0.001	0.469±0.002	0.765±0.002	0.544±0.003
		40	16.04±0	11.57±0.03	4.47±0.03	0.18±0.001	0.386±0.003	0.435±0.002	0.722±0.002	0.459±0.004
		50	16.05±0	11.35±0.05	4.7±0.05	0.182±0.004	0.391±0.003	0.427±0.005	0.707±0.003	0.435±0.007
		60	16.07±0	11.15±0.03	4.91±0.03	0.192±0.003	0.391±0.002	0.418±0.003	0.694±0.002	0.406±0.004
		80	16.08±0	10.83±0.02	5.25±0.02	0.199±0.002	0.392±0.003	0.409±0.004	0.674±0.001	0.369±0.003
		100	16.09±0	10.6±0.08	5.49±0.08	0.209±0.005	0.395±0.004	0.395±0.008	0.659±0.005	0.338±0.01
		200	16.1±0	9.77±0.03	6.33±0.03	0.234±0.002	0.394±0.002	0.373±0.002	0.607±0.002	0.244±0.003
	chr18	2.5	16.57±0	14.05±0.02	2.52±0.02	0.135±0.002	0.299±0.002	0.567±0.004	0.848±0.001	0.712±0.003
		5	17.04±0	13.95±0.01	3.08±0.01	0.152±0.001	0.33±0.001	0.518±0.001	0.819±0.001	0.652±0.002
		10	17.29±0	13.42±0.02	3.86±0.02	0.178±0.003	0.353±0.002	0.469±0.003	0.777±0.001	0.561±0.004
		20	17.42±0	12.57±0.07	4.85±0.07	0.205±0.006	0.364±0.002	0.431±0.006	0.721±0.004	0.449±0.011
		40	17.49±0	11.72±0.05	5.77±0.05	0.228±0.005	0.371±0.003	0.4±0.005	0.67±0.003	0.351±0.008
		50	17.5±0	11.37±0.04	6.13±0.04	0.237±0.004	0.373±0.003	0.391±0.007	0.65±0.002	0.315±0.006
		60	17.51±0	11.16±0.08	6.35±0.08	0.238±0.004	0.374±0.002	0.388±0.004	0.637±0.005	0.295±0.01
		80	17.52±0	10.76±0.07	6.76±0.07	0.252±0.003	0.375±0.002	0.373±0.004	0.614±0.004	0.253±0.006
		100	17.53±0	10.5±0.08	7.03±0.08	0.251±0.006	0.376±0.001	0.372±0.006	0.599±0.005	0.233±0.01
		200	17.54±0	9.86±0.08	7.69±0.08	0.275±0.006	0.381±0.002	0.344±0.007	0.562±0.005	0.171±0.009
Test dataset	chr1	2.5	181.47±0	177.16±0.02	4.31±0.02	0.019±0	0.444±0.002	0.536±0.002	0.976±0	0.958±0
		5	191.64±0	186.76±0.02	4.88±0.02	0.02±0	0.436±0.001	0.544±0.002	0.975±0	0.955±0

3		10	197.8±0	192.15±0.02	5.65±0.02	0.022±0	0.437±0.002	0.542±0.002	0.971±0	0.949±0
		20	201.33±0	194.59±0.06	6.74±0.06	0.025±0	0.445±0.002	0.53±0.002	0.967±0	0.94±0.001
		40	203.24±0	194.48±0.18	8.76±0.18	0.032±0.001	0.466±0.004	0.502±0.005	0.957±0.001	0.922±0.002
		50	203.63±0	193.76±0.18	9.86±0.18	0.036±0.001	0.474±0.005	0.49±0.004	0.952±0.001	0.912±0.002
		60	203.89±0	192.94±0.1	10.96±0.1	0.04±0.001	0.48±0.005	0.479±0.006	0.946±0	0.901±0.001
		80	204.23±0	191.37±0.34	12.86±0.34	0.044±0.002	0.489±0.004	0.466±0.004	0.937±0.002	0.884±0.004
		100	204.43±0	189.8±0.18	14.63±0.18	0.05±0.001	0.493±0.004	0.457±0.004	0.928±0.001	0.866±0.002
		200	204.84±0	182.89±0.79	21.95±0.79	0.066±0.006	0.506±0.006	0.428±0.006	0.893±0.004	0.796±0.01
		400	205.04±0	171.22±0.83	33.82±0.83	0.091±0.007	0.515±0.008	0.394±0.011	0.835±0.004	0.68±0.011
	chr2	2.5	132.22±0	128.82±0.01	3.4±0.01	0.02±0	0.431±0.002	0.549±0.002	0.974±0	0.954±0
		5	138.31±0	134.46±0.01	3.85±0.01	0.02±0	0.424±0.003	0.556±0.003	0.972±0	0.951±0
		10	141.79±0	137.39±0.03	4.4±0.03	0.021±0	0.426±0.001	0.552±0.001	0.969±0	0.945±0
		20	143.71±0	138.37±0.08	5.34±0.08	0.024±0.001	0.438±0.005	0.538±0.005	0.963±0.001	0.934±0.001
		40	144.73±0	137.5±0.16	7.23±0.16	0.03±0.001	0.465±0.004	0.505±0.005	0.95±0.001	0.91±0.002
		50	144.93±0	136.78±0.07	8.16±0.07	0.033±0.001	0.475±0.004	0.491±0.004	0.944±0	0.898±0.001
		60	145.07±0	136.02±0.05	9.05±0.05	0.036±0.001	0.482±0.003	0.482±0.003	0.938±0	0.887±0.001
		80	145.25±0	134.34±0.26	10.9±0.26	0.04±0.003	0.489±0.003	0.471±0.002	0.925±0.002	0.863±0.004
		100	145.36±0	132.75±0.28	12.61±0.28	0.044±0.001	0.498±0.006	0.458±0.006	0.913±0.002	0.841±0.004
		200	145.57±0	125.28±0.64	20.29±0.64	0.063±0.003	0.511±0.004	0.426±0.002	0.861±0.004	0.74±0.009
		400	145.68±0	113.96±0.73	31.72±0.73	0.092±0.005	0.515±0.002	0.393±0.004	0.782±0.005	0.59±0.011
	chr3	2.5	129.43±0	126.36±0.01	3.07±0.01	0.019±0	0.431±0.001	0.549±0.001	0.976±0	0.955±0
		5	134.99±0	131.53±0.01	3.45±0.01	0.02±0	0.423±0.002	0.557±0.002	0.974±0	0.951±0
		10	138.17±0	134.24±0.03	3.93±0.03	0.022±0.001	0.426±0.003	0.553±0.004	0.972±0	0.945±0
		20	139.92±0	135.21±0.02	4.71±0.02	0.025±0	0.44±0.003	0.535±0.003	0.966±0	0.935±0
		40	140.84±0	134.5±0.09	6.34±0.09	0.03±0	0.47±0.005	0.5±0.005	0.955±0.001	0.912±0.001
		50	141.03±0	133.68±0.28	7.35±0.28	0.035±0.003	0.477±0.004	0.488±0.003	0.948±0.002	0.897±0.004
		60	141.16±0	133.07±0.1	8.09±0.1	0.036±0.002	0.482±0.002	0.482±0.004	0.943±0.001	0.887±0.002
		80	141.32±0	131.7±0.16	9.62±0.16	0.041±0.003	0.492±0.005	0.467±0.006	0.932±0.001	0.865±0.003
		100	141.42±0	129.69±0.56	11.73±0.56	0.049±0.003	0.495±0.004	0.456±0.007	0.917±0.004	0.833±0.009
		200	141.61±0	122.35±1.01	19.27±1.01	0.069±0.006	0.512±0.003	0.419±0.009	0.864±0.007	0.722±0.017
		400	141.71±0	111.64±0.77	30.07±0.77	0.101±0.008	0.522±0.006	0.377±0.005	0.788±0.005	0.559±0.016
	chr4	2.5	138.7±0	135.31±0.01	3.38±0.01	0.019±0	0.433±0.001	0.548±0.001	0.976±0	0.954±0
		5	144.47±0	140.66±0.01	3.81±0.01	0.019±0	0.424±0	0.557±0	0.974±0	0.95±0
		10	147.74±0	143.4±0.03	4.33±0.03	0.019±0.001	0.423±0.003	0.557±0.003	0.971±0	0.945±0
		20	149.52±0	144.21±0.04	5.3±0.04	0.022±0.001	0.44±0.003	0.538±0.003	0.965±0	0.933±0.001

		40	150.44±0	143.02±0.16	7.42±0.16	0.028±0.001	0.469±0.004	0.502±0.005	0.951±0.001	0.906±0.002
		50	150.63±0	142.21±0.2	8.42±0.2	0.031±0.002	0.479±0.006	0.49±0.006	0.944±0.001	0.893±0.003
		60	150.76±0	141.2±0.14	9.56±0.14	0.035±0.001	0.484±0.006	0.481±0.006	0.937±0.001	0.878±0.002
		80	150.92±0	139.29±0.35	11.63±0.35	0.039±0.003	0.489±0.008	0.472±0.008	0.923±0.002	0.851±0.005
		100	151.01±0	137.93±0.23	13.08±0.23	0.041±0.002	0.498±0.007	0.461±0.007	0.913±0.002	0.832±0.003
		200	151.21±0	129.14±1.12	22.06±1.12	0.062±0.004	0.512±0.002	0.426±0.004	0.854±0.007	0.713±0.016
		400	151.3±0	116.54±0.53	34.76±0.53	0.095±0.004	0.533±0.008	0.372±0.006	0.77±0.004	0.543±0.009
	chr5	2.5	101.71±0	99.35±0	2.36±0	0.019±0	0.444±0.001	0.538±0.001	0.977±0	0.959±0
		5	105.77±0	103.04±0.02	2.72±0.02	0.02±0.001	0.442±0.002	0.539±0.003	0.974±0	0.955±0
		10	108.16±0	104.92±0.03	3.24±0.03	0.022±0.001	0.446±0.004	0.532±0.004	0.97±0	0.947±0.001
		20	109.5±0	105.4±0.05	4.09±0.05	0.025±0	0.467±0.005	0.508±0.005	0.963±0	0.934±0.001
		40	110.22±0	104.09±0.11	6.14±0.11	0.035±0.001	0.495±0.006	0.47±0.006	0.944±0.001	0.899±0.002
		50	110.37±0	103.52±0.15	6.86±0.15	0.036±0.001	0.505±0.007	0.459±0.007	0.938±0.001	0.888±0.003
		60	110.47±0	102.59±0.22	7.88±0.22	0.04±0.001	0.505±0.004	0.455±0.004	0.929±0.002	0.87±0.004
		80	110.6±0	101.15±0.18	9.46±0.18	0.044±0.001	0.511±0.007	0.445±0.008	0.915±0.002	0.844±0.003
		100	110.68±0	99.86±0.17	10.82±0.17	0.047±0.002	0.515±0.003	0.437±0.003	0.902±0.002	0.822±0.003
		200	110.83±0	93.61±0.69	17.22±0.69	0.064±0.002	0.524±0.006	0.412±0.006	0.845±0.006	0.714±0.011
		400	110.91±0	85.58±0.68	25.33±0.68	0.093±0.007	0.541±0.007	0.366±0.006	0.772±0.006	0.575±0.015
	chr6	2.5	148.9±0	145.39±0.01	3.51±0.01	0.019±0	0.437±0.002	0.545±0.002	0.976±0	0.961±0
		5	156.42±0	152.43±0.01	3.99±0.01	0.019±0	0.428±0.002	0.553±0.001	0.974±0	0.957±0
		10	160.69±0	156.12±0.02	4.57±0.02	0.02±0.001	0.429±0.002	0.551±0.002	0.972±0	0.952±0
		20	163.01±0	157.54±0.03	5.47±0.03	0.024±0	0.443±0.001	0.533±0.002	0.966±0	0.943±0
		40	164.23±0	157.02±0.06	7.21±0.06	0.028±0.001	0.469±0.002	0.503±0.002	0.956±0	0.925±0.001
		50	164.47±0	156.34±0.09	8.13±0.09	0.031±0.001	0.478±0.005	0.492±0.004	0.951±0.001	0.916±0.001
		60	164.64±0	155.57±0.22	9.07±0.22	0.036±0.001	0.485±0.003	0.48±0.003	0.945±0.001	0.905±0.002
		80	164.85±0	154.32±0.28	10.53±0.28	0.038±0.002	0.493±0.002	0.469±0.004	0.936±0.002	0.89±0.003
		100	164.97±0	152.22±0.28	12.75±0.28	0.044±0.002	0.497±0.004	0.459±0.004	0.923±0.002	0.866±0.003
		200	165.23±0	145.37±0.67	19.86±0.67	0.06±0.002	0.514±0.001	0.425±0.002	0.88±0.004	0.787±0.008
		400	165.35±0	134.25±0.56	31.1±0.56	0.085±0.003	0.518±0.007	0.397±0.005	0.812±0.003	0.662±0.007
	chr7	2.5	118.72±26.9	115.33±26.06	3.39±0.84	0.022±0.001	0.451±0.015	0.527±0.016	0.972±0.001	0.947±0.002
		5	136.21±0	132±0.06	4.21±0.06	0.023±0	0.448±0.006	0.529±0.007	0.969±0	0.942±0.001
		10	139.26±0	134.41±0.02	4.85±0.02	0.024±0	0.452±0.004	0.524±0.004	0.965±0	0.935±0
		20	140.9±0	135±0.1	5.9±0.1	0.028±0.001	0.464±0.002	0.508±0.003	0.958±0.001	0.921±0.002
		40	141.76±0	133.79±0.09	7.97±0.09	0.035±0.001	0.481±0.005	0.483±0.004	0.944±0.001	0.893±0.001
		50	141.93±0	133±0.15	8.93±0.15	0.038±0.001	0.494±0.004	0.468±0.004	0.937±0.001	0.88±0.002

		60	142.05±0	132±0.36	10.05±0.36	0.042±0.002	0.497±0.007	0.461±0.008	0.929±0.003	0.864±0.005
		80	142.2±0	130.14±0.17	12.06±0.17	0.045±0.002	0.504±0.006	0.451±0.008	0.915±0.001	0.837±0.002
		100	142.28±0	128.39±0.28	13.9±0.28	0.05±0.003	0.507±0.002	0.443±0.003	0.902±0.002	0.812±0.005
		200	142.46±0	120.88±0.53	21.58±0.53	0.068±0.003	0.515±0.007	0.417±0.005	0.849±0.004	0.705±0.009
		400	142.55±0	108.93±0.46	33.63±0.46	0.099±0.004	0.53±0.004	0.371±0.002	0.764±0.003	0.537±0.009
	chr8	2.5	148.86±0	145.22±0.01	3.64±0.01	0.02±0	0.417±0.001	0.562±0.001	0.976±0	0.952±0
		5	155.52±0	151.5±0.02	4.02±0.02	0.021±0	0.406±0.002	0.573±0.003	0.974±0	0.949±0
		10	159.22±0	154.77±0.01	4.45±0.01	0.02±0.001	0.401±0.002	0.579±0.002	0.972±0	0.945±0
		20	161.2±0	155.97±0.06	5.23±0.06	0.022±0	0.416±0.003	0.562±0.004	0.968±0	0.936±0.001
		40	162.21±0	155.26±0.19	6.95±0.19	0.027±0.002	0.452±0.006	0.521±0.008	0.957±0.001	0.915±0.003
		50	162.42±0	154.75±0.24	7.67±0.24	0.03±0.002	0.46±0.006	0.51±0.007	0.953±0.001	0.906±0.003
		60	162.56±0	153.84±0.16	8.72±0.16	0.033±0.001	0.475±0.003	0.492±0.002	0.946±0.001	0.893±0.002
		80	162.73±0	152.5±0.15	10.23±0.15	0.036±0.001	0.482±0.011	0.482±0.011	0.937±0.001	0.874±0.002
		100	162.83±0	151.08±0.19	11.75±0.19	0.04±0.002	0.494±0.004	0.465±0.005	0.928±0.001	0.855±0.003
		200	163.04±0	144.18±0.45	18.86±0.45	0.055±0.002	0.512±0.003	0.433±0.004	0.884±0.003	0.765±0.006
		400	163.15±0	132.52±0.52	30.62±0.52	0.083±0.004	0.539±0.006	0.378±0.003	0.812±0.003	0.613±0.008
	chr9	2.5	64.51±0	62.93±0.01	1.58±0.01	0.024±0.001	0.43±0.001	0.547±0.002	0.975±0	0.959±0
		5	67.3±0	65.51±0.01	1.79±0.01	0.024±0	0.424±0.002	0.552±0.002	0.973±0	0.955±0
		10	68.95±0	66.91±0.02	2.04±0.02	0.027±0.001	0.423±0.003	0.55±0.003	0.97±0	0.95±0.001
		20	69.88±0	67.43±0.06	2.45±0.06	0.031±0.004	0.433±0.004	0.535±0.005	0.965±0.001	0.94±0.002
		40	70.37±0	67.04±0.07	3.32±0.07	0.039±0.003	0.462±0.003	0.5±0.003	0.953±0.001	0.918±0.002
		50	70.47±0	66.82±0.12	3.64±0.12	0.04±0.006	0.474±0.005	0.487±0.007	0.948±0.002	0.91±0.004
		60	70.53±0	66.33±0.15	4.2±0.15	0.047±0.008	0.476±0.008	0.477±0.009	0.94±0.002	0.895±0.006
		80	70.62±0	65.62±0.26	4.99±0.26	0.051±0.003	0.487±0.007	0.462±0.004	0.929±0.004	0.875±0.007
		100	70.67±0	65.06±0.12	5.61±0.12	0.052±0.004	0.487±0.005	0.46±0.009	0.921±0.002	0.86±0.004
		200	70.77±0	61.52±0.3	9.25±0.3	0.065±0.005	0.499±0.015	0.436±0.015	0.869±0.004	0.768±0.009
		400	70.82±0	56.3±0.56	14.52±0.56	0.084±0.007	0.51±0.007	0.406±0.005	0.795±0.008	0.638±0.017
	chr10	2.5	112.86±0	110.06±0.01	2.8±0.01	0.022±0.001	0.434±0.001	0.544±0.001	0.975±0	0.954±0
		5	116.41±0	113.25±0.01	3.17±0.01	0.022±0.001	0.428±0.002	0.55±0.003	0.973±0	0.949±0
		10	118.33±0	114.57±0.03	3.76±0.03	0.024±0.001	0.433±0.002	0.543±0.002	0.968±0	0.94±0.001
		20	119.33±0	114.49±0.11	4.84±0.11	0.028±0.001	0.448±0.004	0.524±0.004	0.959±0.001	0.923±0.002
		40	119.84±0	112.76±0.06	7.09±0.06	0.034±0.002	0.479±0.003	0.487±0.004	0.941±0.001	0.887±0.001
		50	119.95±0	111.42±0.14	8.52±0.14	0.04±0.002	0.489±0.007	0.471±0.006	0.929±0.001	0.863±0.003
		60	120.02±0	110.33±0.41	9.68±0.41	0.044±0.003	0.493±0.006	0.462±0.009	0.919±0.003	0.844±0.007
		80	120.1±0	108.36±0.47	11.74±0.47	0.047±0.003	0.502±0.002	0.45±0.004	0.902±0.004	0.811±0.008

		100	120.15±0	106.46±0.45	13.69±0.45	0.051±0.003	0.508±0.003	0.441±0.003	0.886±0.004	0.78±0.008
		200	120.26±0	97.75±0.49	22.51±0.49	0.075±0.005	0.521±0.007	0.404±0.007	0.813±0.004	0.635±0.009
		400	120.31±0	86.78±0.27	33.53±0.27	0.101±0.003	0.529±0.003	0.369±0.005	0.721±0.002	0.463±0.004
	chr11	2.5	93.82±0	91.6±0	2.22±0	0.02±0.001	0.428±0.001	0.552±0.001	0.976±0	0.957±0
		5	97.61±0	95.07±0.02	2.54±0.02	0.021±0.001	0.422±0.003	0.558±0.003	0.974±0	0.953±0
		10	99.73±0	96.78±0.02	2.95±0.02	0.021±0	0.423±0.002	0.556±0.002	0.97±0	0.946±0
		20	100.86±0	97.07±0.03	3.79±0.03	0.024±0.001	0.444±0.001	0.532±0.002	0.962±0	0.931±0.001
		40	101.44±0	95.95±0.16	5.49±0.16	0.031±0.002	0.469±0.004	0.5±0.005	0.946±0.002	0.9±0.003
		50	101.55±0	95.2±0.17	6.35±0.17	0.036±0.003	0.478±0.002	0.486±0.004	0.937±0.002	0.883±0.004
		60	101.63±0	94.42±0.22	7.21±0.22	0.039±0.003	0.486±0.003	0.475±0.005	0.929±0.002	0.867±0.005
		80	101.73±0	92.83±0.21	8.9±0.21	0.044±0.003	0.492±0.004	0.464±0.006	0.912±0.002	0.836±0.005
		100	101.79±0	91.4±0.39	10.39±0.39	0.048±0.003	0.497±0.005	0.455±0.005	0.898±0.004	0.808±0.008
		200	101.91±0	85.99±0.33	15.92±0.33	0.068±0.004	0.503±0.005	0.429±0.006	0.844±0.003	0.701±0.006
		400	101.97±0	77.64±0.56	24.32±0.56	0.095±0.003	0.524±0.001	0.382±0.004	0.761±0.005	0.544±0.011
	chr12	2.5	76.27±0	74.31±0.01	1.96±0.01	0.02±0	0.425±0.002	0.554±0.002	0.974±0	0.953±0
		5	79.16±0	76.89±0.01	2.28±0.01	0.021±0.001	0.423±0.002	0.555±0.003	0.971±0	0.947±0
		10	80.78±0	78.05±0.02	2.73±0.02	0.024±0.001	0.434±0.005	0.542±0.005	0.966±0	0.938±0.001
		20	81.64±0	78.03±0.14	3.61±0.14	0.031±0.004	0.459±0.008	0.51±0.011	0.956±0.002	0.917±0.004
		40	82.08±0	76.69±0.09	5.39±0.09	0.039±0.002	0.482±0.002	0.479±0.004	0.934±0.001	0.876±0.002
		50	82.17±0	76.1±0.22	6.08±0.22	0.043±0.005	0.485±0.006	0.472±0.004	0.926±0.003	0.859±0.007
		60	82.23±0	75.31±0.17	6.92±0.17	0.046±0.004	0.499±0.01	0.455±0.011	0.916±0.002	0.84±0.005
		80	82.31±0	73.91±0.25	8.4±0.25	0.051±0.004	0.5±0.006	0.449±0.007	0.898±0.003	0.806±0.006
		100	82.35±0	72.61±0.32	9.74±0.32	0.055±0.003	0.505±0.01	0.441±0.01	0.882±0.004	0.774±0.008
		200	82.44±0	67.27±0.75	15.17±0.75	0.072±0.004	0.515±0.012	0.413±0.009	0.816±0.009	0.649±0.019
		400	82.49±0	60.6±0.91	21.9±0.91	0.098±0.003	0.519±0.01	0.383±0.008	0.735±0.011	0.494±0.021
	chr13	2.5	159.41±0	155.55±0.01	3.86±0.01	0.017±0	0.432±0.001	0.551±0.001	0.976±0	0.955±0
		5	144.16±53.6	140.4±52.31	3.76±1.37	0.017±0.002	0.424±0.001	0.559±0.002	0.974±0.001	0.952±0
		10	173.23±0	168.25±0.02	4.98±0.02	0.018±0	0.423±0.001	0.559±0.001	0.971±0	0.947±0
		20	176.04±0	170.12±0.07	5.92±0.07	0.02±0	0.431±0.005	0.549±0.005	0.966±0	0.938±0.001
		40	177.51±0	169.87±0.1	7.64±0.1	0.023±0.001	0.456±0.004	0.521±0.004	0.957±0.001	0.92±0.001
		50	177.81±0	169.36±0.18	8.46±0.18	0.026±0.001	0.463±0.001	0.511±0.002	0.952±0.001	0.911±0.002
		60	178.02±0	168.76±0.18	9.26±0.18	0.03±0.001	0.468±0.003	0.502±0.003	0.948±0.001	0.902±0.002
		80	178.27±0	167.24±0.3	11.03±0.3	0.035±0.001	0.476±0.002	0.489±0.003	0.938±0.002	0.882±0.003
		100	178.42±0	165.98±0.26	12.44±0.26	0.037±0.002	0.486±0.006	0.478±0.008	0.93±0.001	0.867±0.003
		200	178.73±0	159.04±0.31	19.69±0.31	0.056±0.002	0.504±0.007	0.44±0.008	0.89±0.002	0.785±0.004

		400	178.89±0	148.7±1.17	30.19±1.17	0.083±0.004	0.527±0.003	0.39±0.005	0.831±0.007	0.663±0.014
	chr14	2.5	141.3±0	137.98±0.01	3.32±0.01	0.02±0	0.428±0.002	0.552±0.002	0.977±0	0.954±0
		5	147.95±0	144.18±0.02	3.77±0.02	0.02±0	0.423±0.002	0.557±0.002	0.975±0	0.95±0
		10	151.64±0	147.26±0.06	4.38±0.06	0.022±0.001	0.428±0.004	0.55±0.004	0.971±0	0.943±0.001
		20	153.59±0	148.07±0.07	5.52±0.07	0.028±0	0.449±0.003	0.523±0.003	0.964±0	0.929±0.001
		40	154.59±0	146.93±0.07	7.66±0.07	0.036±0.002	0.475±0.002	0.489±0.003	0.95±0	0.9±0.001
		50	154.8±0	146.12±0.13	8.68±0.13	0.038±0.001	0.484±0.006	0.478±0.006	0.944±0.001	0.887±0.002
		60	154.93±0	145.24±0.22	9.69±0.22	0.043±0.002	0.482±0.005	0.476±0.003	0.937±0.001	0.872±0.003
		80	155.11±0	143.52±0.34	11.58±0.34	0.048±0.004	0.489±0.005	0.463±0.006	0.925±0.002	0.846±0.006
		100	155.21±0	141.8±0.19	13.41±0.19	0.052±0.002	0.501±0.005	0.448±0.005	0.914±0.001	0.822±0.003
		200	155.42±0	134.87±0.3	20.55±0.3	0.07±0.002	0.518±0.004	0.413±0.003	0.868±0.002	0.724±0.005
		400	155.52±0	123.84±0.67	31.69±0.67	0.104±0.002	0.532±0.008	0.364±0.009	0.796±0.004	0.565±0.008
	chr15	2.5	133.47±0	130.26±0	3.22±0	0.019±0	0.444±0.002	0.537±0.002	0.976±0	0.956±0
		5	139.55±0	135.96±0.02	3.58±0.02	0.019±0	0.433±0.004	0.549±0.004	0.974±0	0.954±0
		10	142.88±0	138.93±0.02	3.96±0.02	0.019±0	0.428±0.002	0.554±0.002	0.972±0	0.95±0
		20	144.65±0	139.96±0.02	4.69±0.02	0.021±0.001	0.438±0.003	0.541±0.003	0.968±0	0.941±0
		40	145.57±0	139.38±0.12	6.19±0.12	0.027±0.001	0.46±0.003	0.514±0.004	0.957±0.001	0.922±0.002
		50	145.76±0	138.72±0.13	7.03±0.13	0.029±0.002	0.472±0.002	0.498±0.002	0.952±0.001	0.911±0.002
		60	145.88±0	138.05±0.17	7.84±0.17	0.032±0.002	0.476±0.005	0.492±0.005	0.946±0.001	0.901±0.002
		80	146.04±0	136.68±0.1	9.36±0.1	0.038±0.002	0.485±0.004	0.476±0.002	0.936±0.001	0.88±0.002
		100	146.14±0	135.26±0.39	10.88±0.39	0.041±0.001	0.493±0.003	0.466±0.003	0.926±0.003	0.861±0.005
		200	146.33±0	128.31±0.78	18.02±0.78	0.06±0.006	0.513±0.004	0.428±0.006	0.877±0.005	0.766±0.013
		400	146.43±0	117.9±0.85	28.53±0.85	0.086±0.004	0.53±0.009	0.384±0.008	0.805±0.006	0.626±0.012
	chr16	2.5	88.73±0	86.43±0.01	2.3±0.01	0.023±0.001	0.425±0.001	0.552±0.001	0.974±0	0.955±0
		5	92.33±0	89.74±0.02	2.59±0.02	0.023±0.001	0.417±0.004	0.56±0.004	0.972±0	0.951±0
		10	94.37±0	91.41±0.02	2.97±0.02	0.025±0	0.42±0.003	0.555±0.003	0.969±0	0.945±0
		20	95.47±0	91.78±0.06	3.69±0.06	0.032±0.001	0.434±0.005	0.534±0.005	0.961±0.001	0.931±0.001
		40	96.05±0	90.79±0.21	5.25±0.21	0.038±0.003	0.46±0.004	0.503±0.006	0.945±0.002	0.902±0.004
		50	96.16±0	90.05±0.09	6.11±0.09	0.044±0.003	0.468±0.007	0.488±0.009	0.936±0.001	0.885±0.002
		60	96.24±0	89.61±0.24	6.63±0.24	0.045±0.003	0.475±0.002	0.48±0.003	0.931±0.003	0.875±0.005
		80	96.34±0	88.01±0.22	8.33±0.22	0.053±0.005	0.478±0.004	0.469±0.005	0.913±0.002	0.841±0.006
		100	96.4±0	86.6±0.36	9.8±0.36	0.061±0.004	0.481±0.004	0.458±0.004	0.898±0.004	0.811±0.007
		200	96.52±0	80.25±0.25	16.27±0.25	0.089±0.005	0.491±0.007	0.42±0.007	0.831±0.003	0.68±0.008
		400	96.58±0	72.97±0.31	23.61±0.31	0.112±0.002	0.502±0.005	0.386±0.005	0.756±0.003	0.539±0.007
	chr17	2.5	83.78±0	81.56±0.01	2.23±0.01	0.024±0.001	0.448±0.001	0.528±0.001	0.973±0	0.951±0

		5	86.78±0	84.23±0.01	2.55±0.01	0.025±0.001	0.445±0.003	0.53±0.003	0.971±0	0.946±0
		10	88.43±0	85.46±0.05	2.97±0.05	0.027±0.001	0.453±0.003	0.52±0.004	0.966±0.001	0.938±0.001
		20	89.31±0	85.61±0.08	3.7±0.08	0.032±0.002	0.469±0.004	0.5±0.005	0.959±0.001	0.922±0.002
		40	89.77±0	84.75±0.09	5.02±0.09	0.037±0.003	0.492±0.005	0.471±0.006	0.944±0.001	0.895±0.003
		50	89.86±0	84.13±0.22	5.74±0.22	0.039±0.003	0.504±0.007	0.457±0.006	0.936±0.002	0.88±0.005
		60	89.93±0	83.66±0.18	6.27±0.18	0.04±0.003	0.509±0.003	0.451±0.004	0.93±0.002	0.869±0.004
		80	90±0	82.36±0.18	7.64±0.18	0.045±0.002	0.514±0.006	0.441±0.008	0.915±0.002	0.839±0.004
		100	90.05±0	81.15±0.32	8.9±0.32	0.049±0.003	0.522±0.01	0.429±0.008	0.901±0.004	0.813±0.007
		200	90.14±0	75.96±0.23	14.18±0.23	0.063±0.002	0.53±0.01	0.407±0.01	0.843±0.003	0.702±0.005
		400	90.19±0	68.67±0.31	21.52±0.31	0.086±0.004	0.534±0.005	0.38±0.006	0.761±0.003	0.549±0.008
	chr18	2.5	60.34±0	58.83±0.01	1.51±0.01	0.023±0.001	0.438±0.005	0.54±0.005	0.975±0	0.956±0
		5	62.8±0	61.09±0.01	1.71±0.01	0.024±0.001	0.429±0.004	0.547±0.005	0.973±0	0.952±0
		10	64.21±0	62.28±0.01	1.93±0.01	0.025±0.001	0.429±0.003	0.546±0.003	0.97±0	0.947±0
		20	64.98±0	62.7±0.05	2.27±0.05	0.027±0.002	0.434±0.006	0.539±0.004	0.965±0.001	0.938±0.002
		40	65.38±0	62.41±0.04	2.97±0.04	0.034±0.002	0.46±0.01	0.506±0.009	0.955±0.001	0.918±0.001
		50	65.46±0	62.06±0.09	3.4±0.09	0.036±0.002	0.473±0.001	0.492±0.004	0.948±0.001	0.906±0.003
		60	65.52±0	61.67±0.12	3.85±0.12	0.039±0.004	0.477±0.014	0.484±0.017	0.941±0.002	0.893±0.004
		80	65.59±0	60.98±0.18	4.61±0.18	0.042±0.001	0.491±0.005	0.467±0.005	0.93±0.003	0.872±0.005
		100	65.63±0	60.28±0.19	5.35±0.19	0.048±0.003	0.483±0.006	0.469±0.005	0.918±0.003	0.85±0.006
		200	65.71±0	56.46±0.44	9.25±0.44	0.066±0.004	0.484±0.007	0.449±0.004	0.859±0.007	0.738±0.014
		400	65.75±0	51.41±0.79	14.35±0.79	0.09±0.011	0.494±0.011	0.416±0.011	0.782±0.012	0.592±0.028

Table S4. GWAS datasets and candidate genes reported in the original paper and identified using imputed-genotypes.

Traits	Samples	Phenotypes	Associations (candidate genes)	
			[1]Original report	[2]Imputed-genotypes
[3]Growth and fatness traits	N=1,067,<br>Duroc pig breed,<br>60K & 80K,<br>[38]	D100	*VPS4B*<br>***PHLPP1***<br>***CDH20***<br>***ENSSSCG00000034988***<br>***ENSSSCG00000004911***	*PHLPP1*<br>*ENSSSCG00000034988*<br>*ENSSSCG00000004911*<br>***TNFRSF11A***<br>***MC4R***<br>***CCBE1***<br>***LMAN1***<br>***CPLX4***<br>***ENSSSCG00000048150***<br>***ENSSSCG00000039179***<br>***ENSSSCG00000041383***<br>***ENSSSCG00000048538***<br>***ENSSSCG00000050691***<br>***ENSSSCG00000045579***<br>***ENSSSCG00000047436***
		L100	***ADAMTS2***	*ADAMTS2*<br>***MAML1***<br>***CANX***<br>***HNRNPH1***<br>***RUFY1***<br>***ZNF879***<br>***GRM6***<br>***ZNF454***<br>***ZFP2***<br>***ZNF354B***<br>***PROP1***<br>***CLK4***<br>***COL23A1***<br>***RNF44***<br>***CLTB***<br>***ssc-mir-1271***<br>***ARL10***<br>***KIAA1191***<br>***SIMC1***<br>***THOC3***<br>***SFXN1***<br>***TMEM171***<br>***ENSSSCG00000046532***<br>***ENSSSCG00000044467***<br>***ENSSSCG00000041652***<br>***ENSSSCG00000034785***<br>***ENSSSCG00000042891***<br>***ENSSSCG00000042242***<br>***ENSSSCG00000051188***
		B100	***GRM4***<br>***NUDT3***<br>***SNRPC***<br>*TXN2*<br>*TSHZ1*<br>*PHLLP1*<br>*CDH20*<br>*GRM4*<br>*ENSSSCG00000034988*<br>*ENSSSCG00000004911*	*GRM4*<br>*NUDT3*<br>*SNRPC*<br>***MC4R***<br>***TSHZ1***<br>***DXO***<br>***STK19***<br>***PRRT1***<br>***TINAG***<br>***MLIP***<br>***HMGA1***<br>***PACSIN1***<br>***SPDEF***<br>***ILRUN***

				UHRF1BP1<br>***TAF11***<br>***ANKS1A***<br>***ENSSSCG00000037476***<br>***ENSSSCG00000023160***<br>***ENSSSCG00000048538***<br>***ENSSSCG00000050691***<br>***ENSSSCG00000045579***
[3]Reproduction traits	N=1,067,<br>Duroc pig breed,<br>60K & 80K,<br>[39]	LSB	***BICC1***<br>*TNX*<br>*KCNA1*<br>*ZDHHC18*<br>*MAP2K6*	*BICC1*<br>***NCF4***<br>***IFT27***<br>***CACNG2***<br>***ENSSSCG00000000145***<br>***ENSSSCG00000041362***<br>***ENSSSCG00000036761***
		LWB	*FAM135B*<br>*EPHB2*<br>*SEMA4D*	***PEBP4***<br>***PLEK***<br>***CNRIP1***<br>***PNO1***<br>***WDR92***<br>***ZC3H3***<br>***PTK2***<br>***SPATC1***<br>***ENSSSCG00000036115***<br>***ENSSSCG00000034561***<br>***ENSSSCG00000039331***<br>***ENSSSCG00000041622***
		LSW	*TMEM132D*<br>*TBX3*<br>*FAM110A*	-
[4]Growth and fatness traits	N=365,<br>Sujiang pig breed,<br>[40]	BW	*GABRB3*<br>*ZNF106*	-
		BL	-	-
		BH	-	***ENSSSCG00000032052***
		CC	-	-
		CW	-	-
		HW	-	-
		BF	*XKR4*<br>*MGAM*<br>*TAS2R38*	***RABEPK***<br>***LMX1B***<br>***RALGPS1***<br>***ANGPTL2***<br>***LRSAM1***<br>***NIBAN2***<br>***SH2D3C***<br>***ST6GALNAC4***<br>***PIP5KL1***<br>***DPM2***<br>***FAM102A***<br>***CCL8***<br>***ASIC2***<br>***TNXB***<br>***ENSSSCG00000022295***

Notes:

[1]Candidate genes reported in the original paper and re-found with imputed-genotypes were bolded.

[2]Candidate genes identified using imputed-genotypes and novel genes were bolded.

[3]The original study used 32,147 SNPs for GWAS and took the genome-wide suggestive significant threshold as to be 1.08×10^{-4}; imputed-genotypes after using the same

filtrations as in the ordinal study included 10,856,918 SNPs and took the genome-wide suggestive significant threshold as to be 2.05×10^{-6} (1/ 488,357).

[4]The original study used 53,702 SNPs and took the genome-wide significant threshold as to be 9.31×10^{-6}; the imputed-genotype after using the same filtration as in the original study included 19,401,022 SNPs and took the genome-wide suggestive significant threshold as to be 6.46×10^{-7} (1/1,548,065).

D100 = days to 100 kg; L100 = loin muscle area at 100 kg; B100 = backfat thickness at 100 kg; LSB = litter size at birth; LWB= litter weight at birth; LSW = litter size at weaning; BW = body weight; BL = body length; BH = body height; CC = chest circumference; CW = chest width, HW = hip width; BF = backfat thickness.

Table S5. The accuracies (mean ± standard error) of GEBVs of different traits in Xu et al study based on SNP datasets across ten cross-validation replicates.

Trait[1]	GBLUP_CHIP	GBLUP_IMP[b]	GBLUP_PRUNE[2]
BW	0.827 ± 0.003	**0.831 ± 0.003 (↑)**	**0.831 ± 0.003 (↑)**
BL	0.778 ± 0.005	**0.779 ± 0.005 (↑)**	**0.774 ± 0.005 (↓)**
BF	0.780 ± 0.005	**0.782 ± 0.006 (↑)**	**0.788 ± 0.005 (↑)**
CC	0.793 ± 0.004	0.793 ± 0.004 (=)	**0.801 ± 0.004 (↑)**
BH	0.844 ± 0.003	0.840 ± 0.003 (↓)	0.840 ± 0.003 (↓)
CW	0.844 ± 0.003	**0.842 ± 0.003 (↓)**	**0.847 ± 0.003 (↑)**
HW	0.848 ± 0.004	**0.852 ± 0.004 (↑)**	**0.852 ± 0.005 (↑)**

[1]BW = body weight; BL = body length; BF = backfat thickness; BH = body height; CC = chest circumference; CW = chest width, HW = hip width.

[2]The symbols in parentheses represent that the prediction accuracy of GBLUP_IMP or GBLUP_PRUNE is higher than (↑ and bolded), lower than (↓) or equal to (=) the accuracy of GBLUP_CHIP.

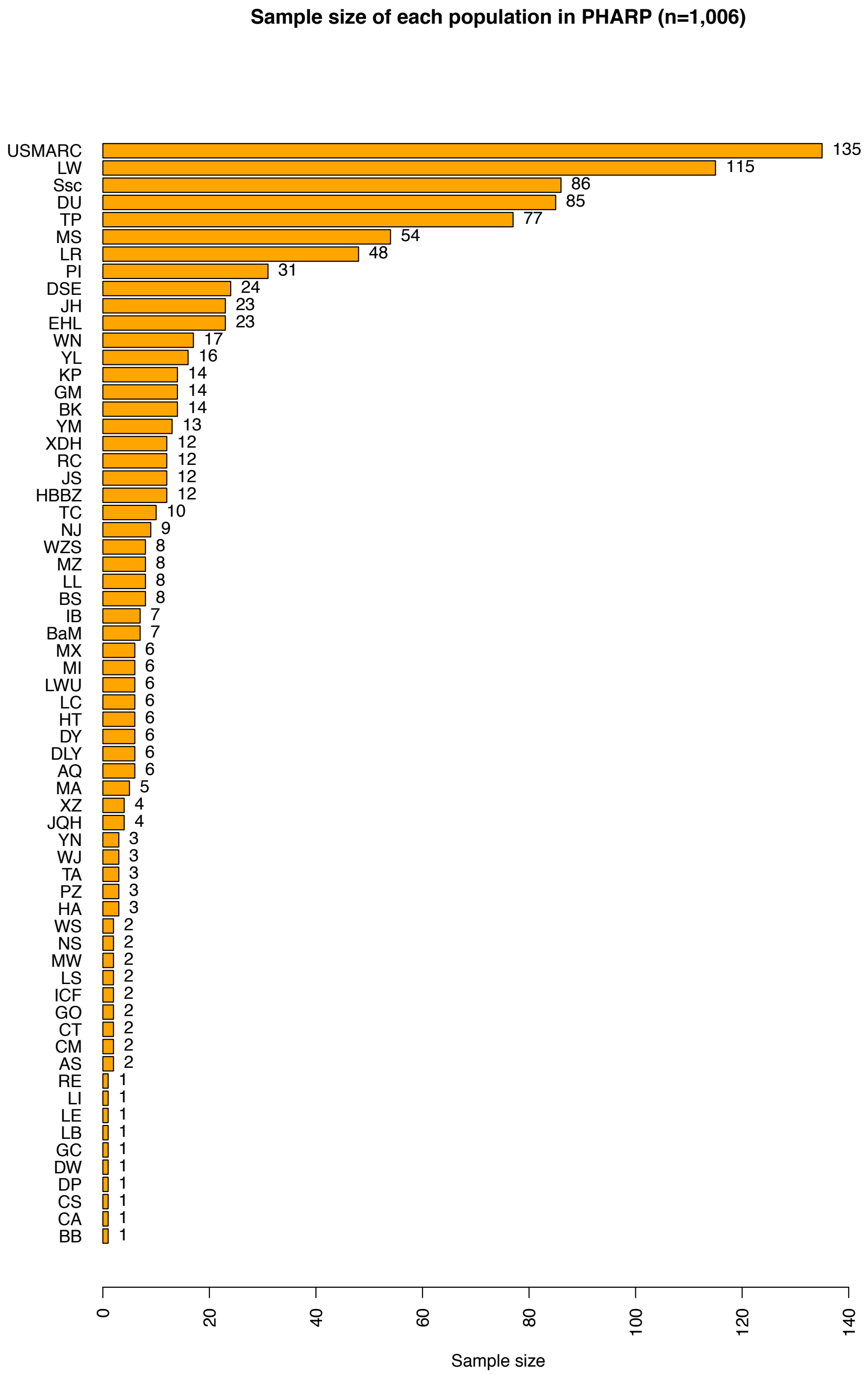

Figure S1. Sample size of each population in PHARP.

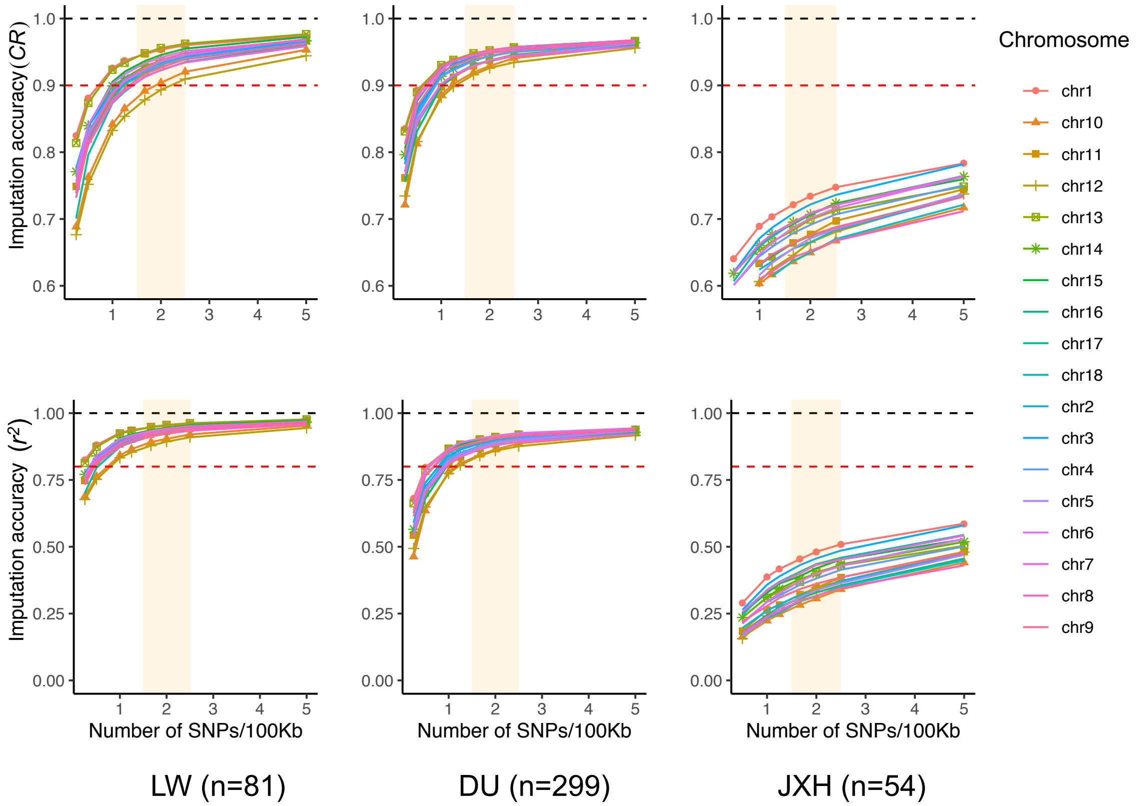

Figure S2. Imputation accuracy estimated by mimicking the imputed panel with different densities (repeated 5 times) of SNPs on chromosomes using three test datasets (see more result detail in Table S4).

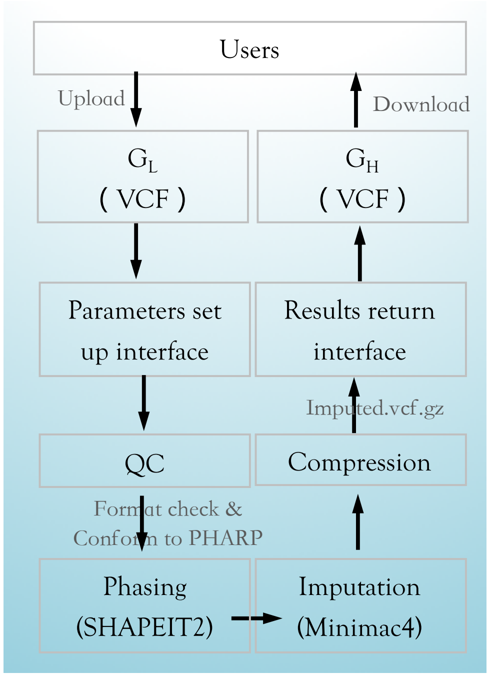

G_L: Low-density genotypes ; G_H: High-density genotypes

Figure S3. The flow chart of the design consisting major steps for PHARP imputation server.

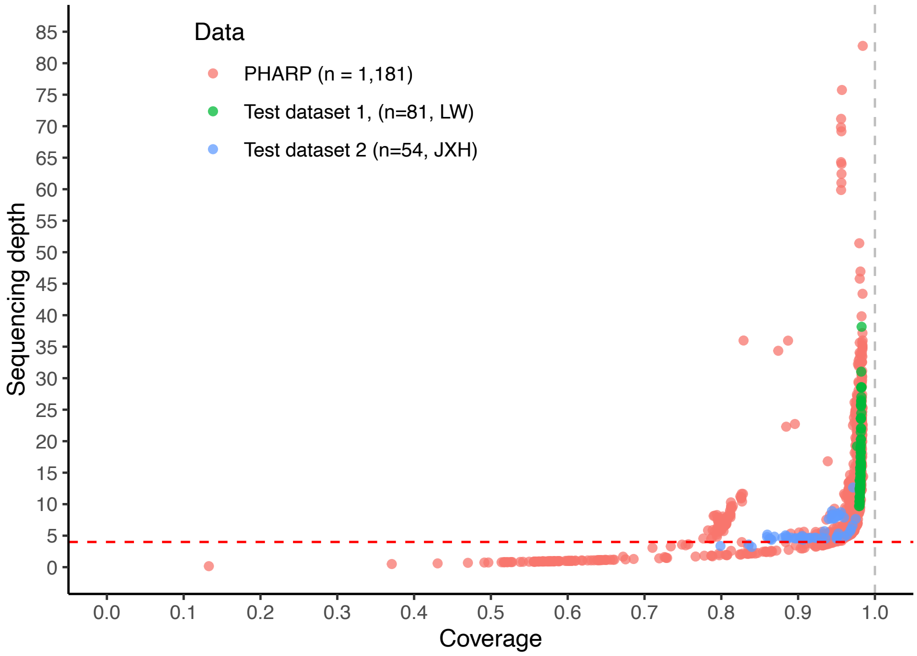

Figure S4. The individual's coverage and sequencing depth distribution among the used datasets. The red dashed line was a depth of 4X, the grey dashed line was a coverage of 1.

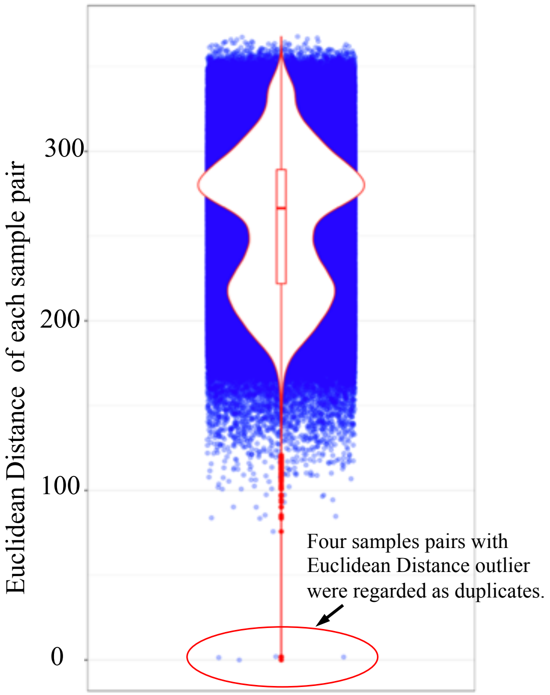

Figure S5. The boxplot of the genetic distance measured by Euclidean distance for each sample pair.

REFERENCE:

1. Bosse M, Megens HJ, Madsen O, Paudel Y, Frantz LA, Schook LB, Crooijmans RP, Groenen MA: **Regions of homozygosity in the porcine genome: consequence of demography and the recombination landscape.** *PLoS Genet* 2012, **8:**e1003100.
2. Bosse M, Megens HJ, Frantz LA, Madsen O, Larson G, Paudel Y, Duijvesteijn N, Harlizius B, Hagemeijer Y, Crooijmans RP, Groenen MA: **Genomic analysis reveals selection for Asian genes in European pigs following human-mediated introgression.** *Nat Commun* 2014, **5:**4392.
3. Rubin CJ, Megens HJ, Barrio AM, Maqbool K, Sayyab S, Schwochow D, Wang C, Carlborg O, Jern P, Jorgensen CB, et al: **Strong signatures of selection in the domestic pig genome.** *Proceedings of the National Academy of Sciences of the United States of America* 2012, **109:**19529-19536.
4. Groenen MAM, Archibald AL, Uenishi H, Tuggle CK, Takeuchi Y, Rothschild MF, Rogel-Gaillard C, Park C, Milan D, Megens HJ, et al: **Analyses of pig genomes provide insight into porcine demography and evolution.** *Nature* 2012, **491:**393-398.
5. Fang X, Mou Y, Huang Z, Li Y, Han L, Zhang Y, Feng Y, Chen Y, Jiang X, Zhao W, et al: **The sequence and analysis of a Chinese pig genome.** *Gigascience* 2012, **1:**16.
6. Vamathevan JJ, Hall MD, Hasan S, Woollard PM, Xu M, Yang YL, Li X, Wang XL, Kenny S, Brown JR, et al: **Minipig and beagle animal model genomes aid species selection in pharmaceutical discovery and development.** *Toxicology and Applied Pharmacology* 2013, **270:**149-157.
7. Li MZ, Tian SL, Jin L, Zhou GY, Li Y, Zhang Y, Wang T, Yeung CKL, Chen L, Ma JD, et al: **Genomic analyses identify distinct patterns of selection in domesticated pigs and Tibetan wild boars.** *Nature Genetics* 2013, **45:**1431-U1180.
8. Wang C, Wang H, Zhang Y, Tang Z, Li K, Liu B: **Genome-wide analysis reveals artificial selection on coat colour and reproductive traits in Chinese domestic pigs.** *Mol Ecol Resour* 2015, **15:**414-424.
9. Choi JW, Chung WH, Lee KT, Cho ES, Lee SW, Choi BH, Lee SH, Lim W, Lim D, Lee YG, et al: **Whole-genome resequencing analyses of five pig breeds, including Korean wild and native, and three European origin breeds.** *DNA Research* 2015, **22:**259-267.
10. Moon S, Kim TH, Lee KT, Kwak W, Lee T, Lee SW, Kim MJ, Cho K, Kim N, Chung WH, et al: **A genome-wide scan for signatures of directional selection in domesticated pigs.** *BMC Genomics* 2015, **16:**130.
11. Ai H, Fang X, Yang B, Huang Z, Chen H, Mao L, Zhang F, Zhang L, Cui L, He W, et al: **Adaptation and possible ancient interspecies introgression in pigs identified by whole-genome sequencing.** *Nat Genet* 2015, **47:**217-225.

12. Li M, Tian S, Yeung CK, Meng X, Tang Q, Niu L, Wang X, Jin L, Ma J, Long K, et al: **Whole-genome sequencing of Berkshire (European native pig) provides insights into its origin and domestication.** *Sci Rep* 2014, **4:**4678.
13. Molnar J, Nagy T, Steger V, Toth G, Marincs F, Barta E: **Genome sequencing and analysis of Mangalica, a fatty local pig of Hungary.** *BMC Genomics* 2014, **15:**761.
14. Ramirez O, Burgos-Paz W, Casas E, Ballester M, Bianco E, Olalde I, Santpere G, Novella V, Gut M, Lalueza-Fox C, et al: **Genome data from a sixteenth century pig illuminate modern breed relationships.** *Heredity (Edinb)* 2015, **114:**175-184.
15. Bosse M, Megens HJ, Madsen O, Crooijmans RP, Ryder OA, Austerlitz F, Groenen MA, de Cara MA: **Using genome-wide measures of coancestry to maintain diversity and fitness in endangered and domestic pig populations.** *Genome Res* 2015, **25:**970-981.
16. Frantz LA, Schraiber JG, Madsen O, Megens HJ, Cagan A, Bosse M, Paudel Y, Crooijmans RP, Larson G, Groenen MA: **Evidence of long-term gene flow and selection during domestication from analyses of Eurasian wild and domestic pig genomes.** *Nat Genet* 2015, **47:**1141-1148.
17. Jeong H, Song KD, Seo M, Caetano-Anolles K, Kim J, Kwak W, Oh JD, Kim E, Jeong DK, Cho S, et al: **Exploring evidence of positive selection reveals genetic basis of meat quality traits in Berkshire pigs through whole genome sequencing.** *BMC Genet* 2015, **16:**104.
18. Kim J, Cho S, Caetano-Anolles K, Kim H, Ryu YC: **Genome-wide detection and characterization of positive selection in Korean Native Black Pig from Jeju Island.** *Bmc Genetics* 2015, **15**.
19. Revilla M, Puig-Oliveras A, Castello A, Crespo-Piazuelo D, Paludo E, Fernandez AI, Ballester M, Folch JM: **A global analysis of CNVs in swine using whole genome sequence data and association analysis with fatty acid composition and growth traits.** *Plos One* 2017, **12**.
20. Zhang YB, Zhang LC, Yue JW, Wei X, Wang LG, Liu X, Gao HM, Hou XH, Zhao FP, Yan H, Wang LX: **Genome-wide identification of RNA editing in seven porcine tissues by matched DNA and RNA high-throughput sequencing.** *Journal of Animal Science and Biotechnology* 2019, **10**.
21. Ma YL, Zhang SX, Zhang KL, Fang CC, Xie SS, Du XY, Li XY, Ni DB, Zhao SH: **Genomic Analysis To Identify Signatures of Artificial Selection and Loci Associated with Important Economic Traits in Duroc Pigs.** *G3-Genes Genomes Genetics* 2018, **8:**3617-3625.
22. Reimer C, Rubin CJ, Sharifi AR, Ha NT, Weigend S, Waldmann KH, Distl O, Pant SD, Fredholm M, Schlather M, Simianer H: **Analysis of porcine body size variation using re-sequencing data of miniature and large pigs.** *Bmc Genomics* 2018, **19**.
23. Keel BN, Nonneman DJ, Rohrer GA: **A survey of single nucleotide polymorphisms identified from whole-genome sequencing and their functional effect in the porcine genome.** *Animal Genetics* 2017, **48:**404-411.

24. Lu MD, Han XM, Ma YF, Irwin DM, Gao Y, Deng JK, Adeola AC, Xie HB, Zhang YP: **Genetic variations associated with six-white-point coat pigmentation in Diannan small-ear pigs.** *Scientific Reports* 2016, **6**.
25. Bianco E, Soto HW, Vargas L, Perez-Enciso M: **The chimerical genome of Isla del Coco feral pigs (Costa Rica), an isolated population since 1793 but with remarkable levels of diversity.** *Molecular Ecology* 2015, **24:**2364-2378.
26. Bianco E, Nevado B, Ramos-Onsins SE, Perez-Enciso M: **A Deep Catalog of Autosomal Single Nucleotide Variation in the Pig.** *Plos One* 2015, **10**.
27. Funkhouser SA, Steibel JP, Bates RO, Raney NE, Schenk D, Ernst CW: **Evidence for transcriptome-wide RNA editing among Sus scrofa PRE-1 SINE elements.** *Bmc Genomics* 2017, **18**.
28. Zhao PJ, Yu Y, Feng W, Du H, Yu J, Kang HM, Zheng XR, Wang ZQ, Liu GE, Ernst CW, et al: **Evidence of evolutionary history and selective sweeps in the genome of Meishan pig reveals its genetic and phenotypic characterization.** *Gigascience* 2018, **7**.
29. Lin Y, Tang QZ, Li Y, He MN, Jin L, Ma JD, Wang X, Long KR, Huang ZQ, Li XW, et al: **Genomic analyses provide insights into breed-of-origin effects from purebreds on three-way crossbred pigs.** *Peerj* 2019, **7**.
30. Heckel T, Schmucki R, Berrera M, Ringshandl S, Badi L, Steiner G, Ravon M, Kung E, Kuhn B, Kratochwil NA, et al: **Functional analysis and transcriptional output of the Gottingen minipig genome.** *Bmc Genomics* 2015, **16**.
31. Keel BN, Nonneman DJ, Lindholm-Perry AK, Oliver WT, Rohrer GA: **A Survey of Copy Number Variation in the Porcine Genome Detected From Whole-Genome Sequence.** *Frontiers in Genetics* 2019, **10**.
32. Li MZ, Chen L, Tian SL, Lin Y, Tang QZ, Zhou XM, Li DY, Yeung CKL, Che TD, Jin L, et al: **Comprehensive variation discovery and recovery of missing sequence in the pig genome using multiple de novo assemblies.** *Genome Research* 2017, **27:**865-874.
33. Falker-Gieske C, Blaj I, Preuss S, Bennewitz J, Thaller G, Tetens J: **GWAS for Meat and Carcass Traits Using Imputed Sequence Level Genotypes in Pooled F2-Designs in Pigs.** *G3-Genes Genomes Genetics* 2019, **9:**2823-2834.
34. Yan GR, Guo TF, Xiao SJ, Zhang F, Xin WS, Huang T, Xu WW, Li YP, Zhang ZY, Huang LS: **Imputation-Based Whole-Genome Sequence Association Study Reveals Constant and Novel Loci for Hematological Traits in a Large-Scale Swine F-2 Resource Population.** *Frontiers in Genetics* 2018, **9**.
35. Zhu YL, Li WB, Yang B, Zhang ZY, Ai HS, Ren J, Huang LS: **Signatures of Selection and Interspecies Introgression in the Genome of Chinese Domestic Pigs.** *Genome Biology and Evolution* 2017, **9:**2592-2603.
36. Chen H, Huang M, Yang B, Wu ZP, Deng Z, Hou Y, Ren J, Huang LS: **Introgression of Eastern Chinese and Southern Chinese haplotypes contributes to the improvement of fertility and immunity in European modern pigs.** *Gigascience* 2020, **9**.
37. Nosková A, Hiltpold M, Janett F, Echtermann T, Fang Z-H, Sidler X, Selige C, Hofer A, Neuenschwander S, Pausch H: **Infertility due to defective sperm flagella caused by an intronic deletion in DNAH17 that perturbs splicing.** *Genetics* 2020, **217**.

38. Zhang Z, Chen Z-t, Diao S-q, Ye S-p, Wang J-y, Gao N, Yuan X-l, Chen Z-m, Zhang H, Li J-q: **Identifying the complex genetic architecture of growth and fatness traits in a Duroc pig population.** *Journal of Integrative Agriculture* 2020, **19**.
39. Zhang Z, Chen Z, Ye S, He Y, Huang S, Yuan X, Chen Z, Zhang H, Li J: **Genome-Wide Association Study for Reproductive Traits in a Duroc Pig Population.** *Animals (Basel)* 2019, **9**.
40. Xu P, Ni L, Tao Y, Ma Z, Hu T, Zhao X, Yu Z, Lu C, Zhao X, Ren J: **Genome-wide association study for growth and fatness traits in Chinese Sujiang pigs.** *Anim Genet* 2020, **51:**314-318.
